# Efficacy and safety of adjunctive therapy to lamotrigine, lithium, or valproate monotherapy in bipolar depression: a systematic review and meta-analysis of randomized controlled trials

**DOI:** 10.1186/s40345-022-00271-7

**Published:** 2022-10-21

**Authors:** Taku Maruki, Tomohiro Utsumi, Masahiro Takeshima, Yu Fujiwara, Marie Matsui, Yumi Aoki, Hiroyuki Toda, Norio Watanabe, Koichiro Watanabe, Yoshikazu Takaesu

**Affiliations:** 1 Department of Neuropsychiatry, Kyorin University School of Medicine, Tokyo, Japan; 2 Department of Psychiatry, The Jikei University School of Medicine, Tokyo, Japan; 3 Department of Neuropsychiatry, Akita University Graduate School of Medicine, Akita, Japan; 4 Department of Psychiatry, School of Medicine, National Defense Medical College, Saitama, Japan; 5 Psychiatric & Mental Health Nursing, Graduate School of Nursing Science, St. Luke’s International University, Tokyo, Japan; 6 Department of Psychiatry, Soseikai General Hospital, Kyoto, Japan; 7 Department of Neuropsychiatry, Faculty of Medicine, University of the Ryukyus, 207 Uehara, Nishihara, Okinawa 903-0215 Japan

**Keywords:** Antipsychotic drug, Bipolar depression, Lamotrigine, Lithium, Treatment-resistant depression

## Abstract

**Background:**

The efficacy and safety of adjunctive therapy are unclear in bipolar depression. In this systematic review and meta-analysis, we aimed to evaluate the efficacy and safety of second-generation antipsychotic, lamotrigine, lithium, or valproate therapy used in adjunction with lamotrigine, lithium, or valproate monotherapy in bipolar depression. A literature search of major electronic databases was conducted in February 2021, and all articles published until then were eligible. Two researchers independently screened relevant publications, extracted data, and evaluated methodological quality according to the Cochrane criteria.

**Results:**

Five studies met the inclusion criteria. The meta-analysis revealed significant differences in the following outcomes: (i) remission rates from depressive episodes (risk ratio [RR]: 1.23, 95% confidence interval [CI] 1.01–1.50, p = 0.04), (ii) improvement in depressive symptoms (standardized mean difference [SMD]: 0.21, 95% CI 0.09–0.34, p = 0.001), (iii) improvement in quality of life (SMD: 0.22, 95% CI 0.06–0.37, p = 0.005), and (iv) rate of adverse events during the study period (RR: 1.12, 95% CI 1.03–1.22, p = 0.008). There was no significant difference between adjunctive therapy and monotherapy in the emergence of suicide-related behaviors, dropout rate during the study period, or rate of manic switching.

**Conclusions:**

Our results suggest that adjunctive second-generation antipsychotics, lamotrigine, lithium, or valproate increase both the benefits and risks in patients with bipolar depression, although there is no significant difference in severe adverse events. Adjunctive therapy should be provided through shared decision-making while considering the patients’ condition in clinical settings.

**Supplementary Information:**

The online version contains supplementary material available at 10.1186/s40345-022-00271-7.

## Background

Compared with manic episodes, bipolar depression imposes a greater burden on patients owing to factors, such as social dysfunction, higher risk of suicide, and longer disease duration (Huxley and Baldessarini 2007; Kupka et al. 2007). Even mild depressive symptoms can cause social dysfunction in patients with bipolar depression (Kauer-Sant'Anna et al. 2009). Furthermore, bipolar depression is associated with a lower quality of life (QOL) and poorer cognitive performance (Khafif et al. 2021; Matsuo et al. 2021). Therefore, the improvement of the symptoms of patients with bipolar depression is important for both patients and society. Regarding medications as the first-line treatment of bipolar depression, several treatment guidelines recommend monotherapy with second-generation antipsychotic drugs (SGA), such as lurasidone and quetiapine, as well as with mood stabilizers (MS), such as lithium (Li) and lamotrigine (LTG) (National Collaborating Centre for Mental Health (UK) 2014; Fountoulakis et al. 2017; Goodwin et al. 2016; Yatham et al. 2018). However, some patients are resistant to monotherapy with SGA or MS in clinical settings regardless of these recommended treatment guidelines. The International College of Neuropsychopharmacology (CINP) guidelines define these patients as having treatment-resistant bipolar depression and recommend other treatment options for their condition (Fountoulakis et al. 2020). There are a few treatment recommendations, including electroconvulsive therapy (ECT), transcranial magnetic stimulation, and bright light therapy, for patients with treatment-resistant bipolar depression. However, transcranial magnetic stimulation and bright light therapy have not shown any clear evidence (Hett and Marwaha 2020; Takeshima et al. 2020). There is evidence that ECT is an effective treatment option for bipolar depression, but it is difficult to conduct ECT for all patients because of the limited resource of the intervention (Schoeyen et al. 2015). Therefore, it is necessary to develop a treatment strategy for bipolar depression.

Adjunctive therapy is expected to be a candidate treatment strategy for bipolar depression. However, adjunctive therapy can increase the risk of potential adverse effects (Galling et al. 2015). Therefore, the clinical question arises as to whether the benefits of adjunctive therapy outweigh the risks when compared with those of monotherapy in bipolar depression. The results of two recent randomized controlled trials (RCTs) have been inconsistent in addressing this question. One study demonstrated the efficacy of adjunctive therapy in improving depressive symptoms (Loebel et al. 2014), whereas the other did not show a significant difference in the improvement of depressive symptoms between adjunctive combination therapy and monotherapy (Suppes et al. 2016). Additionally, many treatment guidelines do not provide clear evidence for both the efficacy and safety of adjunctive therapy with SGA or MS for bipolar depression although these guidelines mention the efficacy of this therapy (National Collaborating Centre for Mental Health (UK) 2014; Fountoulakis et al. 2017; Yatham et al. 2018). Nonetheless, clinicians are likely to use this adjunctive therapy for bipolar depression with inadequate response to monotherapy, even though the efficacy and safety have not been accurately evaluated (Kim et al. 2021). Consequently, there is a significant need for further evidence supporting the use of adjunctive therapy with SGA or MS for bipolar depression with inadequate response to monotherapy.

Therefore, in this systematic review and meta-analysis, we aimed to clarify the efficacy and safety of adjunctive therapy with SGA or MS for bipolar depression.

## Methods

This study was conducted in accordance with the PRISMA recommendations for reporting systematic reviews and meta-analyses (Booth et al. 2012) and was preregistered with PROSPERO (registration number: CRD42021269725) (Liberati et al. 2009) (Additional file 3).

### Search strategy

We searched the electronic databases, PubMed (search date: February 16, 2021), Cochrane Central Register of Controlled Trials (CENTRAL; search date: February 16, 2021), and Embase (search date: February 16, 2021), for RCTs, using appropriate subject headings and relevant search terms (e.g., “bipolar disorder” and “randomized controlled trial;” see Additional file 1: Table S1). When necessary, we contacted the authors of specific studies to clarify additional points.

### Inclusion criteria

Studies meeting the following criteria were included in the review:

1. RCTs performed at the individual or cluster level. Crossover studies were included if they reported results during the first phase of the study (i.e., before the crossover), as the carry-over effect of the first treatment might affect the subsequent phases.
2. Participants diagnosed with bipolar I or II depression, including mixed features and/or rapid cycling, according to the diagnostic criteria used in the specific study (any recognized diagnostic criteria).
3. Participants not taking antidepressants.
4. Studies in which carbamazepine, Li, LTG, and valproic acid (VPA, including divalproex) were used as the MS.
5. Interventions comprised adjunctive therapy with SGA or MS during baseline treatment with SGA or MS.
6. The control groups comprised patients receiving adjunctive therapy with a placebo during baseline treatment with SGA or MS.

### Article selection process

One author (YA) removed duplicates prior to eligibility screening. Subsequently, two groups of screeners, with two authors in each group, were created (Group 1: TM and TU, Group 2: YF and MM). In each group, the two authors independently screened the titles and abstracts of the identified references to exclude irrelevant studies. Subsequently, in each group, the two authors independently evaluated the full texts of these references, and ineligible reports were excluded according to the above criteria. The reasons for exclusion were recorded by the authors in each group. Any disagreement between the screeners was resolved by another author (YT) after thorough and systematic discussions. After identifying eligible studies, the full text of each study was examined.

### Outcome measures

The primary outcome measures included the following: (1) remission rate from depressive symptoms; and (2) improvement in depressive symptoms measured with any validated depressive symptoms assessment tool [e.g., Montgomery Åsberg Depression Rating Scale (MADRS)]. The rates of remission from depressive/mixed episodes were calculated by dividing the number of participants who achieved remission in a group by the total number of participants in that group. When dichotomous outcomes were not reported, but baseline mean, endpoint mean, and corresponding standard deviations of the MADRD (or other depressive symptoms assessment tool) were reported, we converted continuous outcome data expressed as mean and standard deviation into the remission rate, based on a validated method (Furukawa et al. 2005).

When depressive symptoms were reported as a “mean ± standard error,” we converted the value to “mean ± standard deviation.” The secondary outcome measures included the following: (3) improvement in QOL; and (4) rate of adverse events during the study period. We calculated the rate of adverse events during the study period based on the reported adverse events occurring in ≥ 5% of patients in any group. Other outcome measures included; (5) emergence of suicide-related behaviors; (6) dropout rate for all reasons during the study period; and (7) rate of manic switching.

These outcome measures were assessed at baseline and at the endpoint in the study of less than 12 weeks.

### Data extraction, study quality, and risk of bias assessment

Four authors were divided into two groups (Group 1: TM and TU, Group 2: YF and MM) for evaluating the quality of the studies and assessing their risk of bias. The authors in each group carefully and independently extracted the relevant data. Another author (YT) performed checks to ensure the quality and consistency of the assessment. The following variables were extracted from each study: demographics of the participants (e.g., education, employment status, and marital status); diagnostic criteria for bipolar depression; details regarding the participants’ bipolar depression history (type, age of onset, family history, and number of mood episodes); details regarding bipolar depression treatment (e.g., MS, antipsychotics, and antidepressants); concurrent psychiatric disorders; country in which the study was performed; depressive and manic symptoms; and definitions of remission from depressive/mixed episodes and rate of manic switching from depressive or euthymic states, if reported. The following additional variables were also recorded: RCT type, study settings (primary or secondary care), inclusion and exclusion criteria for participant recruitment, contents of the intervention (treatments, timing, and dose), contents of the control group intervention, quality assurance of the intervention, funding source, and number of dropouts from the intervention and control groups. The quality of the included studies was evaluated by the same four authors, divided into the same two groups, using the Cochrane risk of bias assessment tool (Higgins and Green 2011). This assessment evaluates the risk of bias in RCTs in terms of seven domains: (1) random sequence generation, (2) allocation concealment, (3) blinding of participants and personnel, (4) blinding of outcome assessment, (5) incomplete outcome data, (6) selective outcome reporting, and (7) other sources of bias. The rating for each domain can be “yes” (low risk of bias), “no” (high risk of bias), or “unclear” (unclear risk). Any disagreement was resolved through systematic and thorough discussions with another author (YT).

### Statistical analyses

The Cochrane Collaboration Review Manager software (RevMan 5.4) was used for statistical analysis. Continuous outcome data from the intervention and control groups were analyzed using effect sizes [standardized mean differences (SMD)] with 95% confidence intervals (CIs). Dichotomous outcomes were analyzed using risk ratios (RR) with 95% CIs. Random effects models were used in all analyses. Publication bias was evaluated using a funnel plot of the treatment effect against the standard error when at least 10 studies were available (Higgins and Green 2011). Therapeutic effects and adverse events were also assessed. Subgroup analysis was conducted to investigate the sources of heterogeneity. We investigated the difference in effects at each type of combination therapy.

## Results

### Description of studies included in the review

**Fig. 1:**
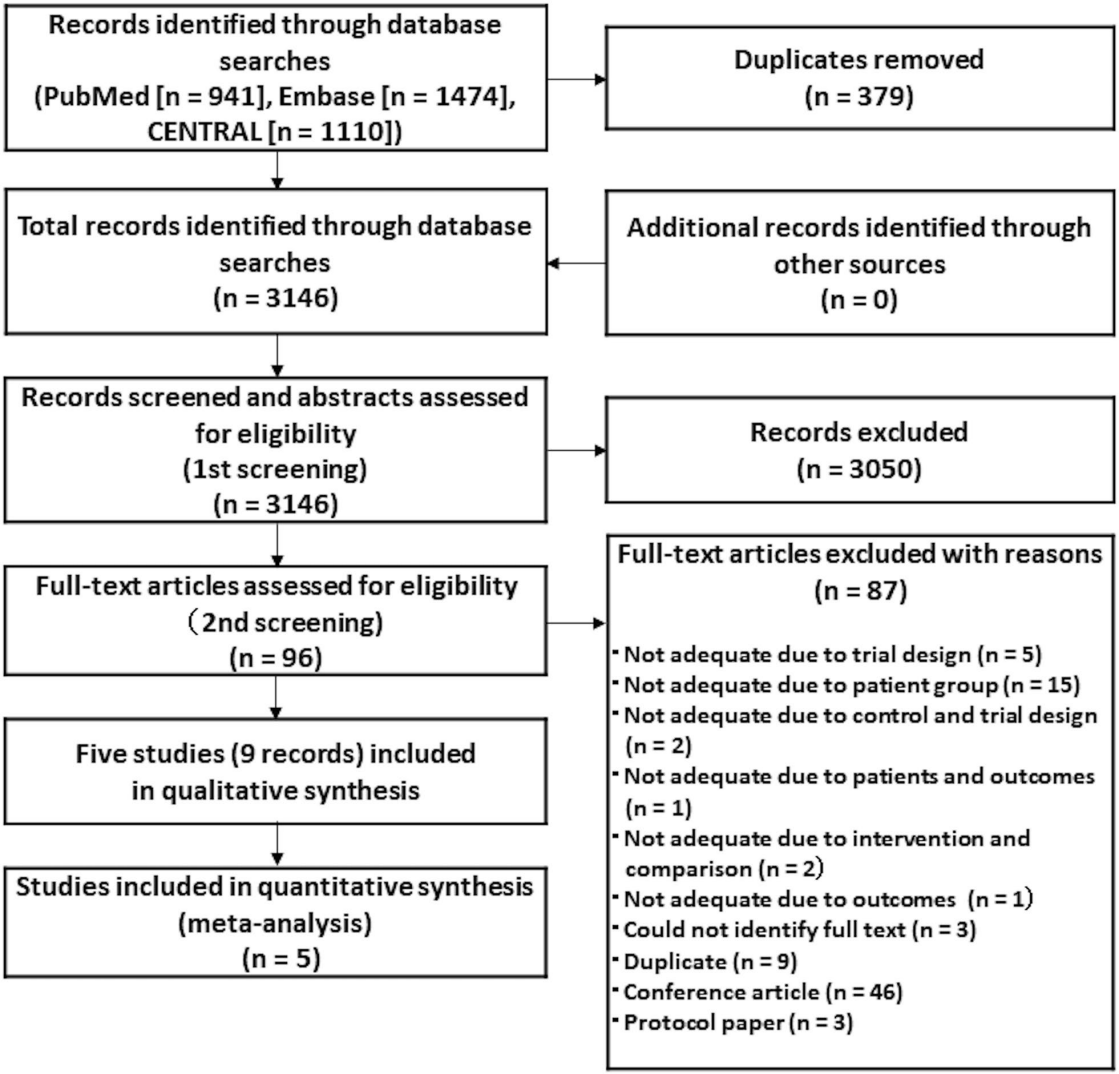
Flowchart of the study selection process

The initial literature search yielded 3146 unique entries published until February 2021 (PubMed, 941; Embase, 1474; and CENTRAL, 1110). After screening the titles and abstracts of the identified reports, the full-text versions of 96 articles were reviewed. Eighty-seven articles were excluded for various reasons (Additional file 1: Table S2), leaving nine articles. The remaining nine articles (five studies) were included in the qualitative synthesis, and five of these RCTs were included in the quantitative synthesis (Fig. 1) (Loebel et al. 2014; Suppes et al. 2016; Loos et al. 2009; Houston et al. 2009; Sachs et al. 2011). The remaining five RCTs investigated the antidepressant effects of adjunctive therapy and monotherapy in treatment-resistant bipolar depression.

### Study characteristics

Nine articles, comprising five studies, published between 2009 and 2016 were included in this review (Loebel et al. 2014; Suppes et al. 2016; Loos et al. 2009, 2011; Houston et al. 2009; Sachs et al. 2011; Citrome et al. 2014; Rajagopalan et al. 2016; Sajatovic et al. 2016). The sample size ranged from 124 to 356, with a total of 1328 participants (Table 1). Of all the participants, 54.7% were female, and the mean age was 41.9 years. The criteria used for the diagnosis of bipolar depression varied across studies. Three studies used the Diagnostic and Statistical Manual of Mental Disorders, Fourth Edition-Text Revision (Loebel et al. 2014; Suppes et al. 2016; Houston et al. 2009). Two studies used the Diagnostic and Statistical Manual of Mental Disorders, Fourth Edition (Loos et al. 2009; Sachs et al. 2011). Four studies defined remission from depressive episodes as a MADRS score of ≤ 12 (Loebel et al. 2014; Suppes et al. 2016; Loos et al. 2009; Sachs et al. 2011). One study defined remission from depressive episodes as a score of ≤ 12 on the Young Mania Rating Scale (YMRS) and a score of ≤ 8 on 21-item Hamilton Depression Rating Scale (HDRS-21) (Houston et al. 2009). Four studies excluded patients with psychotic features (Loebel et al. 2014; Suppes et al. 2016; Loos et al. 2009; Sachs et al. 2011). One study included 4% of patients with psychotic features in the intervention group and 1% in the control group (Houston et al. 2009). An MS was used by 100% of the participants as the baseline treatment. None of the studies permitted the use of antidepressants. Three studies did not permit the use of antipsychotics without intervention therapy (Loos et al. 2009; Houston et al. 2009; Sachs et al. 2011). Two studies did not describe these details (Loebel et al. 2014; Suppes et al. 2016). The percentage of patients who used other permitted concomitant medications was not reported in all the studies. The details of permitted concomitant medications (e.g., benzodiazepines) are presented in Additional file 2: Table S3.

**Table 1:** Characteristics of study participants

Study (year)	Intervention/Control	Age year ± SD	Female n (%)	Type of BD I/II n (%)	Phase of BD	Onset age year ± SD	Rapid cycler n (%)	Taking mood stabilizer n (%)
van der Loos (2009)	Intervention: 64 LTG	45.2 ± 12.1	37/64 (57.8)	I: 43 (67.2) II: 21 (32.8)	MDE	ND	12/64 (18.8)	Li: 64 (100)
	Control: 60 Placebo	47.6 ± 11.6	30/60 (50.0)	I: 41 (68.3) II: 19 (31.7)	MDE	ND	4/64 (6.7)	Li: 60 (100)
Houston (2009)	Intervention: 101 Olanzapine	38.6 ± 11.2	61/101 (60.4)	I: 101 (100)	MD	ND	27/101 (26.7)	VPA: 101 (100)
	Control: 101 Placebo	38.5 ± 11.1	58/101 (57.4)	I: 101 (100)	MD	ND	22/101 (21.8)	VPA: 101 (100)
Sachs (2011)	Intervention: 148 Ziprasidone	40.4 ± 11.4	89/147 (60.5)	I: 148 (100)	MDE	ND	ND	Li: 53 (36.3) VPA: 52 (35.6) LTG: 41 (28.1)
	Control: 150 Placebo	40.4 ± 11.9	91/147 (61.9)	I: 150 (100)	MDE	ND	ND	Li: 54 (36.7) VPA: 52 (35.4) LTG: 41 (27.9)
Loebel (2014)	Intervention: 183 Lurasidone	41.0 ± 11.5	86/179 (48.0)	I: 183 (100)	MDE	28.1 ± 11.0	ND	Li: 90 (50.3) VPA: 89 (49.7)
	Control: 165 Placebo	42.6 ± 11.8	76/161 (47.2)	I: 165 (100)	MDE	29.5 ± 10.7	ND	Li: 73 (45.6) VPA: 87 (54.4)
Suppes (2016)	Intervention: 180 Lurasidone	43.1 ± 11.9	91/176 (51.7)	I: 180 (100)	MDE	28.8 ± 12.1	32/176 (18.2)	Li: 56 (31.8) VPA: 120 (68.2)
	Control: 176 Placebo	44.1 ± 12.0	93/166 (56.0)	I: 176 (100)	MDE	29.8 ± 12.8	21/166 (12.7)	Li: 57 (34.3) VPA: 109 (65.7)

Continuous variable is indicated as mean ± SD. Nominal variable values are indicated as number (%)

BD, bipolar disorder; Li, lithium; LTG, lamotrigine; MDE, major depressive episode; MD, mixed depression; n, number; ND, not described; VPA, valproate, including divalproex; SD, standard deviation

All the studies were individual RCTs (Loebel et al. 2014; Suppes et al. 2016; Loos et al. 2009; Houston et al. 2009; Sachs et al. 2011), which were two-armed and double-blind (participants and assessor) (Loebel et al. 2014; Suppes et al. 2016; Loos et al. 2009; Houston et al. 2009; Sachs et al. 2011). They were all conducted at a primary care facility and provided information regarding financial support (Loebel et al. 2014; Suppes et al. 2016; Loos et al. 2009; Houston et al. 2009; Sachs et al. 2011).

In the intervention group, the intervention drug was added when a patient with bipolar depression did not respond adequately to baseline treatment. All the studies added treatment with SGA or LTG to baseline treatment with LTG, Li, or VPA. There was no study that used SGA and carbamazepine as the baseline treatment. Two studies added lurasidone (dosage range: 20–120 mg/day) to baseline treatment with Li (serum level range: 0.6–1.2 mEq/L) or VPA (50–125 µg/mL) (Loebel et al. 2014; Suppes et al. 2016). One study added olanzapine (5–20 mg/day) to baseline treatment with divalproex (75–125 µg/mL) (Houston et al. 2009). One study added ziprasidone (40–160 mg/day) to baseline treatment with Li (0.6–1.2 mEq/L), VPA (50–125 μg/mL), or LTG (100–200 mg/day) (Sachs et al. 2011). One study added LTG (at week 8: 200 mg/day) to baseline treatment with Li (0.6–1.0 mmol/L) (Loos et al. 2009). In the control group, all the studies added a placebo to each baseline treatment.

### Risk of bias assessment

**Fig. 2:**
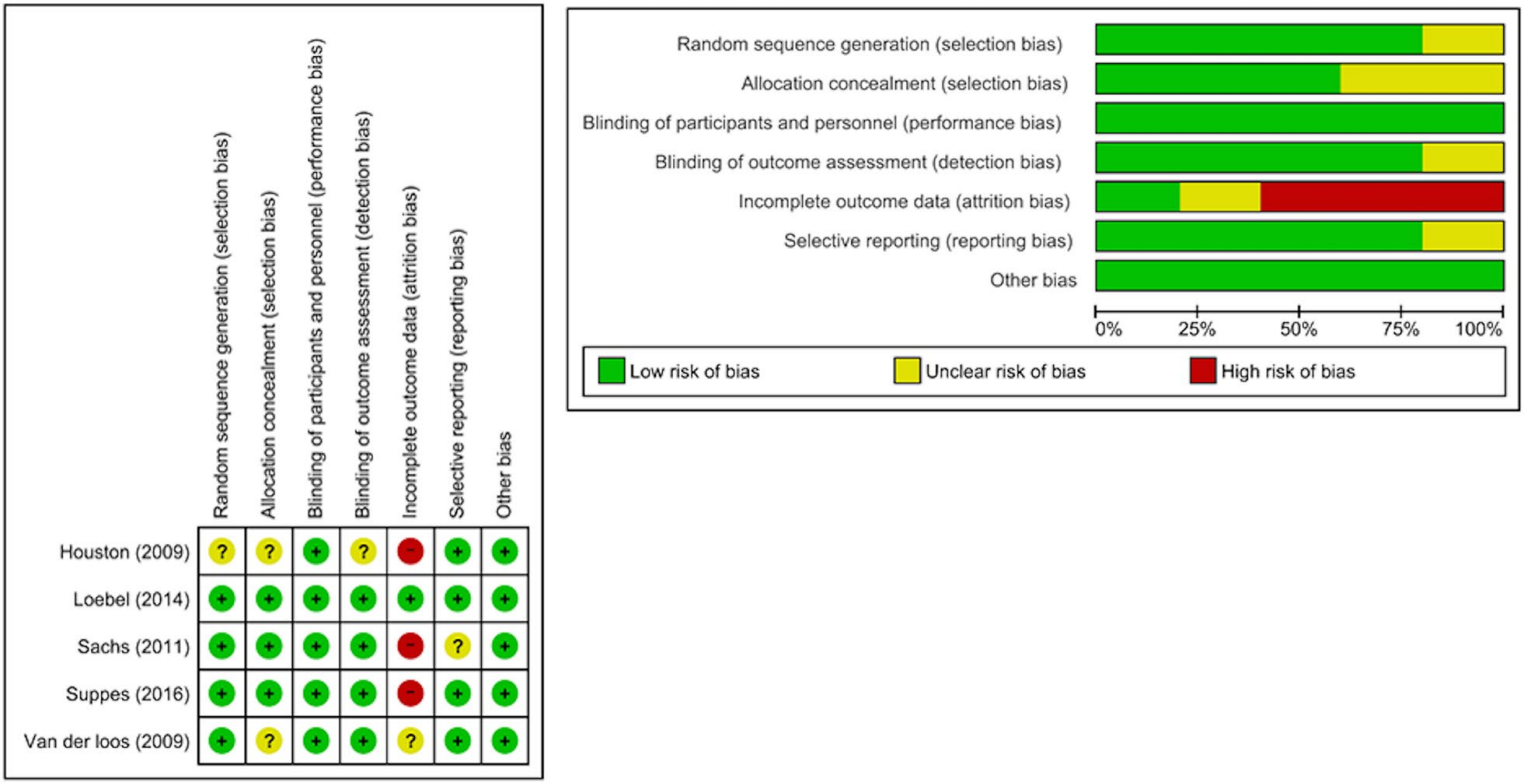
Risk of bias assessment. Green indicates a low risk of bias, yellow indicates an unclear risk of bias, and red indicates a high risk of bias

The risk of bias evaluation (Fig. 2) revealed the following: four RCTs involved adequate randomization methods (Loebel et al. 2014; Suppes et al. 2016; Loos et al. 2009; Sachs et al. 2011); three RCTs reported an adequate allocation concealment procedure (Loebel et al. 2014; Suppes et al. 2016; Sachs et al. 2011); all the RCTs had a low risk of bias in the domain, participant, and personnel blinding (Loebel et al. 2014; Suppes et al. 2016; Loos et al. 2009; Houston et al. 2009; Sachs et al. 2011); and one RCT was judged to have an unclear risk of bias in the domain, blinding of outcome assessments (Houston et al. 2009). With regard to incomplete outcome data, three RCTs had a high risk of bias owing to a high dropout rate (Suppes et al. 2016; Houston et al. 2009; Sachs et al. 2011). One study had unclear reporting bias (Sachs et al. 2011).

### Treatment outcome assessment

The outcomes are summarized in Table 2. Four studies reported remission rates from depressive/mixed episodes (Loebel et al. 2014; Suppes et al. 2016; Houston et al. 2009; Sachs et al. 2011). The remission rate from depressive episodes was reported in all five studies (Loebel et al. 2014; Suppes et al. 2016; Loos et al. 2009; Houston et al. 2009; Sachs et al. 2011). The remission rate from depressive episodes was calculated using depressive symptom scores in only one study (Loos et al. 2009). Improvement in depressive symptoms was reported in all five studies (four studies used MADRS and one study used HDRS-21) (Loebel et al. 2014; Suppes et al. 2016; Loos et al. 2009; Houston et al. 2009; Sachs et al. 2011). Three studies evaluated the improvement in QOL using the Quality of Life, Enjoyment, and Satisfaction Questionnaire (Loebel et al. 2014; Suppes et al. 2016; Sachs et al. 2011). Four studies evaluated the rate of adverse events (Loebel et al. 2014; Suppes et al. 2016; Loos et al. 2009; Sachs et al. 2011), and one of these studies did not describe the details of the adverse events (Houston et al. 2009). Three studies evaluated the emergence of suicide-related behaviors (Loebel et al. 2014; Suppes et al. 2016; Sachs et al. 2011). All studies evaluated the dropout rate for all reasons during the study period (Loebel et al. 2014; Suppes et al. 2016; Loos et al. 2009; Houston et al. 2009; Sachs et al. 2011). Three studies assessed the manic switch rates during the study period (YMRS = 2 and Clinical Global Impressions Scale-Bipolar Version = 1) (Loebel et al. 2014; Suppes et al. 2016; Loos et al. 2009). Attrition was observed in 26.5% of the patients (352/1328).

**Table 2:** Summary of outcomes

Study (year)	Measure for depressive symptoms	Severity of depressive symptoms at baseline: score ± SD		Study duration weeks	Improvement in depressive symptoms score ± SD^a^			Remission rate from depressive symptoms %		
		Intervention	Control		Intervention	Control	P value	Intervention	Control	P value
van der Loos (2009)	MADRS	28.25 ± 5.97	28.82 ± 6.24	8	− 15.38 ± 10.56	− 11.03 ± 10.53	0.024	46.9^a^	30.0^a^	0.0234
Houston (2009)	HDRS-21	22.45 ± 4.2	21.87 ± 4.9	6	− 9.37 ± 5.50	− 7.69 ± 5.43	0.022	31	26	0.437
Sachs (2011)	MADRS	30.0 ± 5.5	28.8 ± 6.1	6	− 13.2 ± 14.4	− 12.9 ± 13.2	0.7921	33.1	37.2	0.5029
Loebel (2014)	MADRS	30.6 ± 5.3	30.8 ± 4.8	6	− 17.1 ± 11.73	− 13.5 ± 11.73	0.005	50	35	0.008
Suppes (2016)	MADRS	29.1 ± 4.9	29.1 ± 4.7	6	− 11.8 ± 9.54	− 10.4 ± 9.54	0.176	34	28	0.227

HDRS-21, 21-item Hamilton Depression Rating Scale; MADRS, Montgomery-Åsberg Depression Rating Scale; SD, standard deviation

^a^Calculated using the data in the paper

**Fig. 3:**
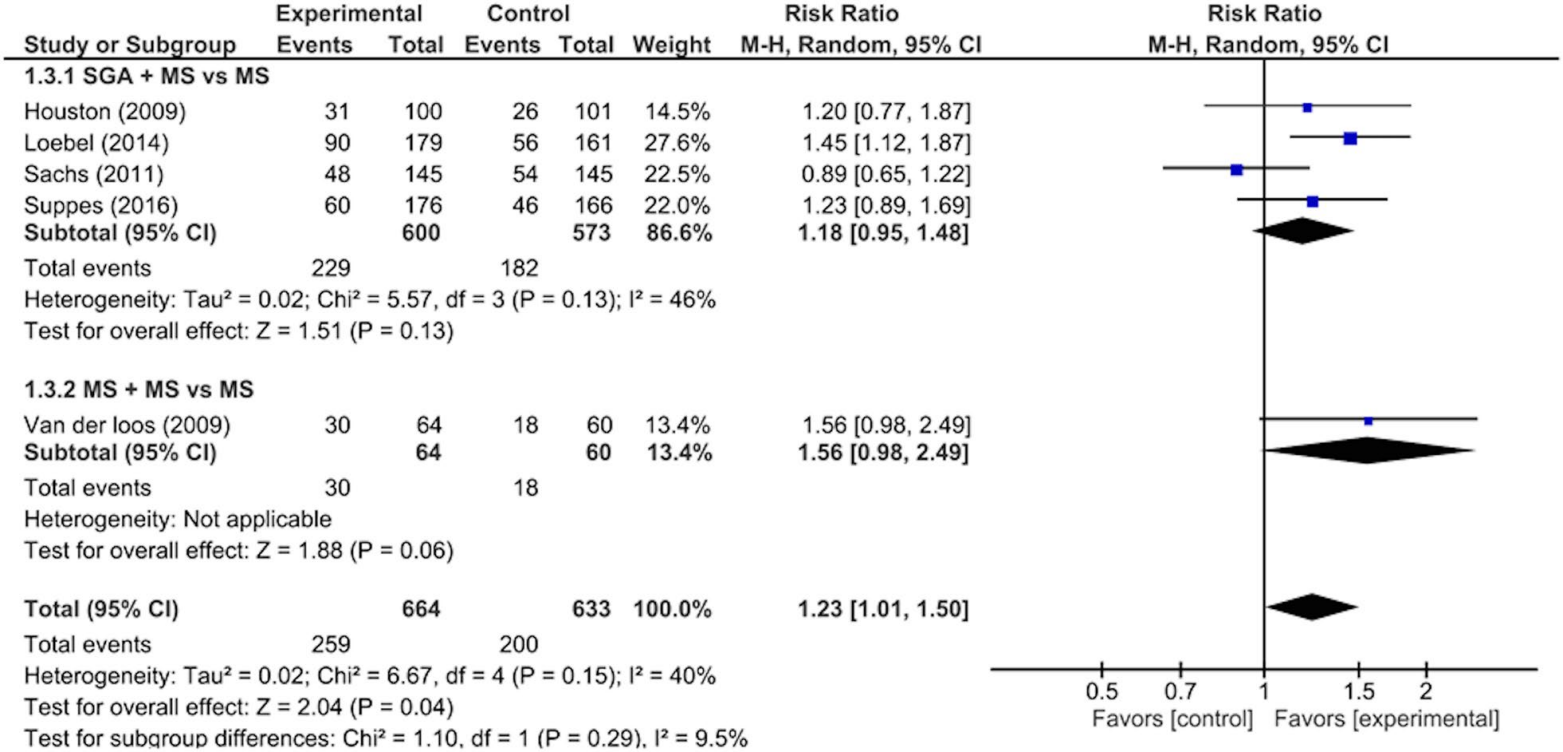
Forest plot of post-intervention treatment effect sizes for remission rate from depressive symptoms. Subgroup analysis of types of combination therapy. CI, confidence interval; SD, standard deviation; MS, mood stabilizers, including lamotrigine, lithium, and valproate; SGA, second-generation antipsychotics

**Fig. 4:**
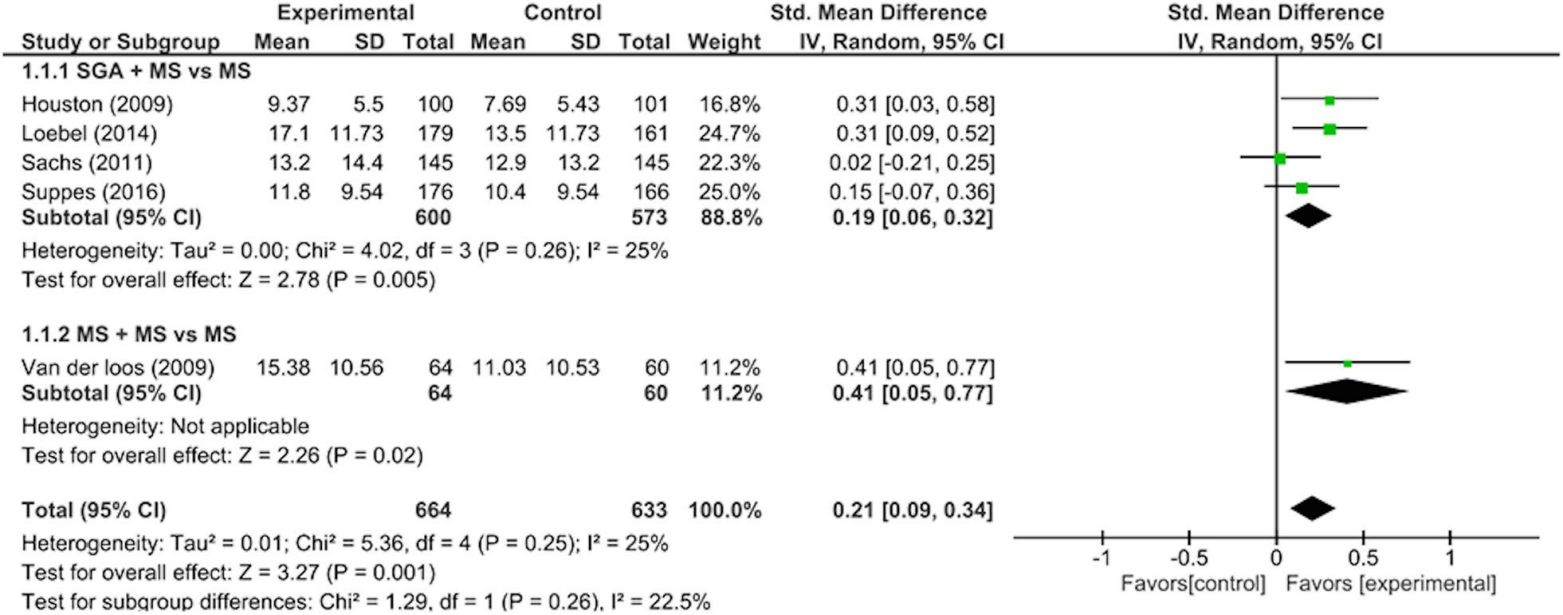
Forest plot of post-intervention treatment effect sizes for improvement in depressive symptoms. Subgroup analysis of types of combination therapy. CI, confidence interval; SD, standard deviation; MS, mood stabilizers, including lamotrigine, lithium, and valproate; SGA, second-generation antipsychotics

**Fig. 5:**
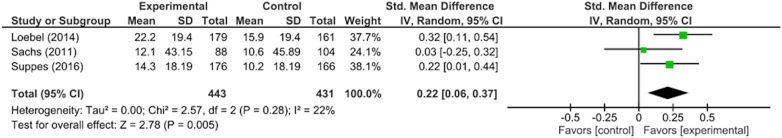
Forest plot of post-intervention treatment effect sizes for improvement in quality of life. CI, confidence interval; SD, standard deviation; MS, mood stabilizers, including lamotrigine, lithium, and valproate; SGA, second-generation antipsychotics

**Fig. 6:**
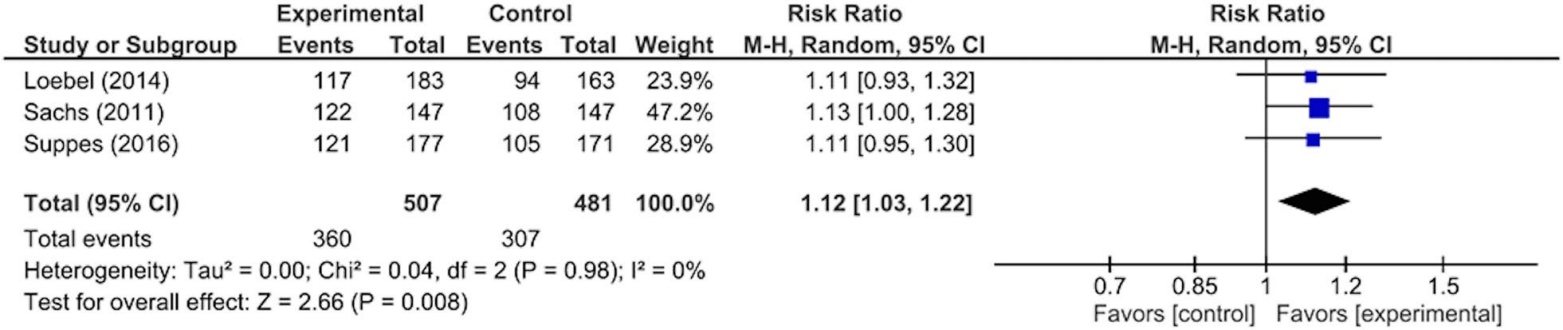
Forest plot of post-intervention treatment effect sizes for rate of adverse events during study period**.** CI, confidence interval; SD, standard deviation; MS, mood stabilizers, including lamotrigine, lithium, and valproate; SGA, second-generation antipsychotics

**Fig. 7:**
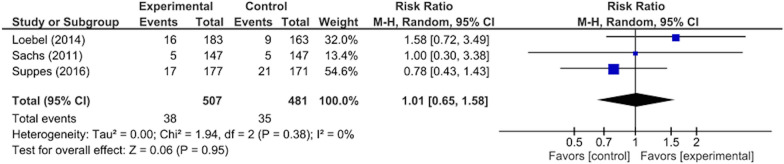
Forest plot of post-intervention treatment effect sizes for emergence of suicide-related behaviors. CI, confidence interval; SD, standard deviation; MS, mood stabilizers, including lamotrigine, lithium, and valproate; SGA, second-generation antipsychotics

**Fig. 8:**
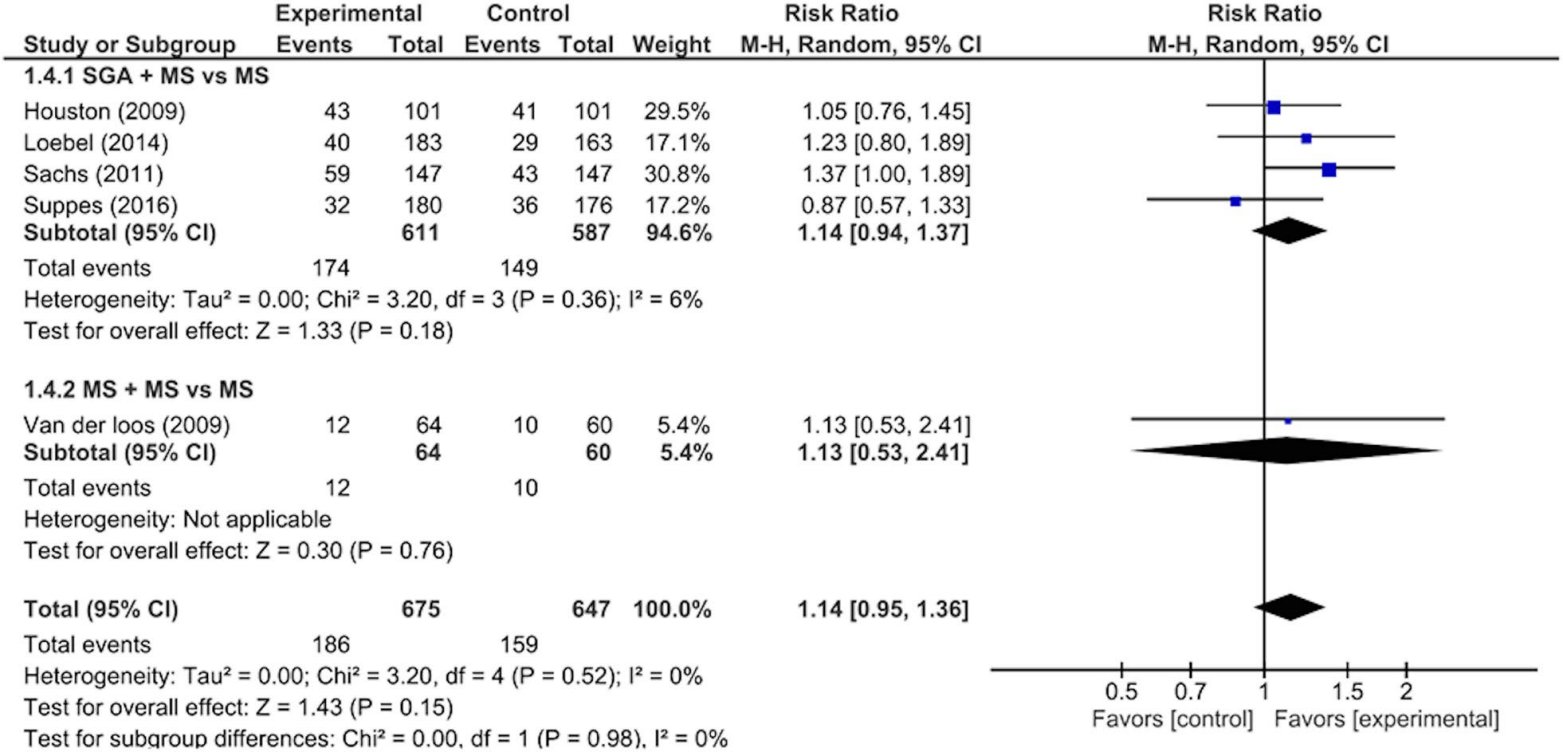
Forest plot of post-intervention treatment effect sizes for dropout rate for all reasons during study period. Subgroup analysis of types of combination therapy. CI, confidence interval; SD, standard deviation; MS, mood stabilizers, including lamotrigine, lithium, and valproate; SGA, second-generation antipsychotics

**Fig. 9:**
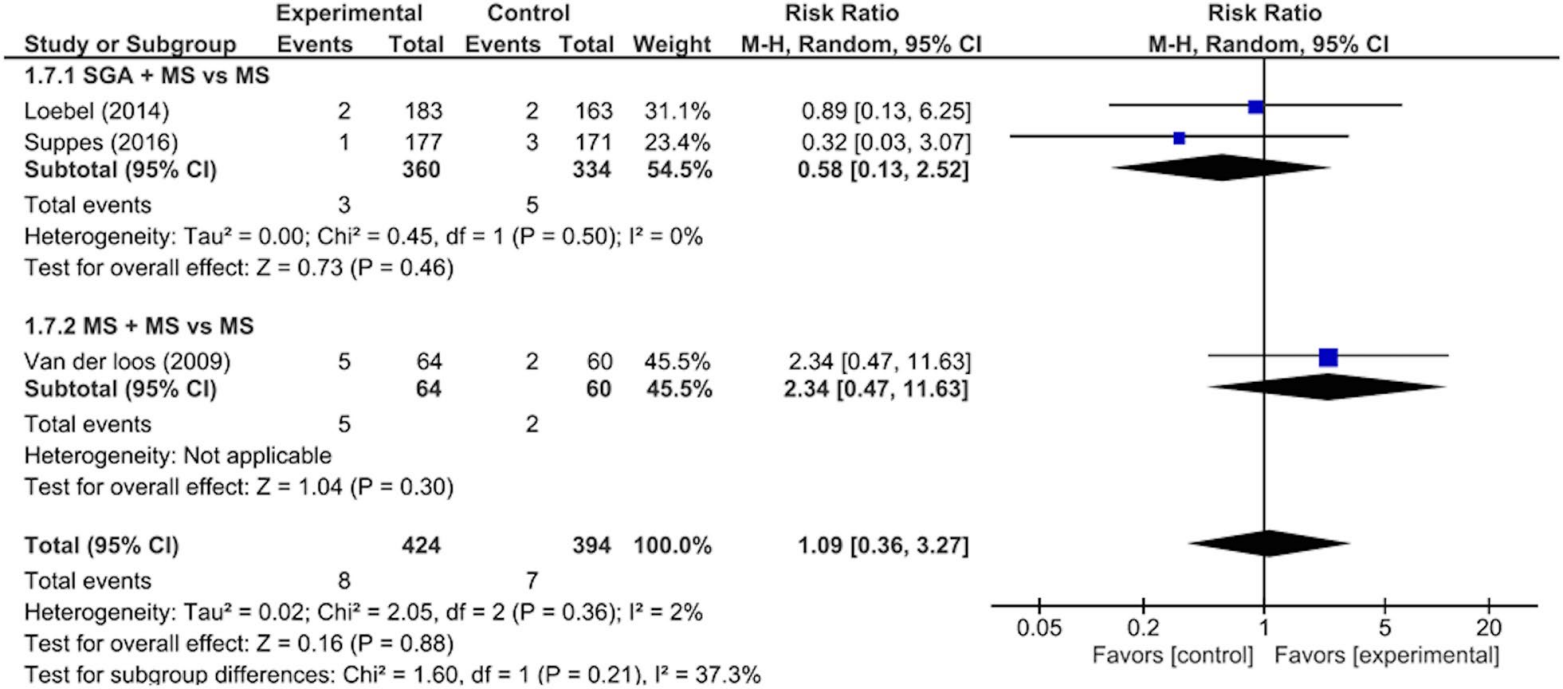
Forest plot of post-intervention treatment effect sizes for rate of manic switching. Subgroup analysis of types of combination therapy. CI, confidence interval; SD, standard deviation; MS, mood stabilizers, including lamotrigine, lithium, and valproate; SGA, second-generation antipsychotics

There were significant differences between the intervention and control groups in the remission rates from depressive/mixed episodes (RR: 1.23, 95% CI 1.01–1.50, p = 0.04; 1297 participants, five studies) (Fig. 3), improvement in depressive symptoms (SMD: 0.21, 95% CI 0.09–0.34, p = 0.001; 1297 participants, five studies) (Fig. 4), improvement in QOL (SMD: 0.22, 95% CI 0.06–0.37, p = 0.005; 874 participants, three studies) (Fig. 5), and rate of adverse events during the study period (RR: 1.12, 95% CI 1.03–1.22, p = 0.008; 988 participants, three studies) (Fig. 6). There was no significant difference between the intervention and control groups in the emergence of suicide-related behaviors (RR: 1.01, 95% CI 0.65–1.58, p = 0.95; 988 participants, three studies) (Fig. 7), dropout rate for all reasons during the study period (RR: 1.14, 95% CI 0.95–1.36, p = 0.15; 1322 participants, five studies) (Fig. 8), and rate of manic switching (RR: 1.09, 95% CI 0.36–3.27, p = 0.88; 818 participants, three studies) (Fig. 9).

Subgroup analysis revealed that when classified according to each type of combination therapy, there were no significant visually and statistically heterogeneity in remission rates from depressive episodes between SGA + MS vs MS + MS (χ^2^ = 1.10, p = 0.29, I^2^ = 9.5%) (Fig. 3). Subgroup analysis revealed that when classified according to each type of combination therapy, there were no significant visually and statistically heterogeneity in the improvement of depressive symptoms between SGA + MS vs MS + MS (χ^2^ = 1.29, p = 0.26, I^2^ = 22.5%) (Fig. 4). Subgroup analysis revealed that when classified according to each type of combination therapy, there were no significant visually and statistically heterogeneity in dropout rates between SGA + MS vs MS + MS (χ^2^ = 0.00, p = 0.98, I^2^ = 0%) (Fig. 8). Subgroup analysis revealed that when classified according to the individual sources of intervention, there was moderate visually heterogeneity, but there was no statistical difference in manic switch rates between SGA + MS vs MS + MS (χ^2^ = 1.60, p = 0.21, I^2^ = 37.3%) (Fig. 9).

## Discussion

To the best of our knowledge, this is the first systematic review and meta-analysis to investigate the efficacy and safety of SGA or MS therapy used in adjunction with SGA or MS monotherapy in bipolar depression. The results show that SGA or MS adjunctive therapy is more effective than placebo in bipolar depression with inadequate response to LTG, Li, or VPA monotherapy. However, in terms of safety, the rate of adverse events was higher with adjunctive therapy than with monotherapy.

Previous studies have reviewed the mechanisms of action of many agents used in bipolar disorder (Goldberg 2019). For example, neither Li nor MS exert their effects directly on serotonin receptors, but they may exert their effects on those receptors indirectly. In SGA, the receptor profiles of each SGA vary, but for example, 5-HT_2A_ antagonism, 5-HT_1A_ receptor partial agonism, α_2b_ receptor antagonism, and D_2_ receptor antagonism might improve bipolar depression in quetiapine, and 5-HT_7_ antagonism might improve bipolar depression in lurasidone (Goldberg 2019; Yatham et al. 2005). The complementary mechanisms of action of each may be effective in adjunctive therapy.

In terms of efficacy, our analysis showed that adjunctive therapy was significantly more effective in improving remission rates, depressive symptoms, and QOL compared with monotherapy. Our results are consistent with the CINP guideline, recommending adjunctive LTG and SGA, such as clozapine and lurasidone, for treatment-resistant bipolar depression (Fountoulakis et al. 2020). Furthermore, our findings are in line with evidence outlined in several treatment guidelines, which describe the efficacy of adjunctive therapy for bipolar depression with inadequate response to monotherapy (National Collaborating Centre for Mental Health (UK) 2014; Fountoulakis et al. 2017; Yatham et al. 2018).

However, the CINP guideline recommend only the use of clozapine, lurasidone, olanzapine, quetiapine, and LTG as adjunctive SGA or MS (Fountoulakis et al. 2020). Similarly, the interventions included in our analysis only involved treatment with lurasidone, olanzapine, ziprasidone, and LTG. Furthermore, the baseline treatment included in our analysis only involved treatment with LTG, Li, and VPA. In light of these findings, although adjunctive SGA and MS may be effective in patients with bipolar depression, who do not respond to MS, the combinations of agents used are probably limited. In addition, considering each of the studies included in this meta-analysis, the study with ziprasidone as an intervention had negative results (Sachs et al. 2011). Except for ziprasidone, all the interventions and baseline medications included in this meta-analysis received relatively high recommendations as monotherapy for the treatment of bipolar depression in many guidelines (National Collaborating Centre for Mental Health (UK) 2014; Fountoulakis et al. 2017; Goodwin et al. xxxx; Yatham et al. 2018; Fountoulakis et al. 2020). This fact suggests that combinations of medications that are highly recommended for bipolar depression as monotherapy may be more effective for bipolar depression. Further studies are needed to identify appropriate combinations among antipsychotics and/or MS.

Regarding safety, a recent network meta-analysis on the efficacy and tolerability of pharmacological monotherapy for acute bipolar depression reported that there were no significant differences in dropout rates for all reasons between patients receiving SGA and MS (apart from aripiprazole) monotherapy and those receiving placebos (Bahji et al. 2020). In line with these results, our meta-analysis also found no significant differences in dropout rates for all reasons between adjunctive therapy and monotherapy, even though dropout rates are usually expected to be higher with adjunctive therapy than with monotherapy. Furthermore, our meta-analysis found no significant differences in the emergence of suicide-related behaviors and rate of manic switching with adjunctive therapy than with monotherapy. Therefore, adjunctive therapy may be considered relatively safe. However, our meta-analysis also showed that adjunctive therapy significantly increased the adverse events compared to monotherapy. Therefore, clinicians should carefully monitor adverse events and consider the risk/benefit of adjunctive therapy in each patient.

We were not able to derive any conclusion regarding the long-term efficacy and safety of adjunctive therapy because there were no long-term RCTs that fulfilled our study criteria. There have been three open-label long-term follow-up studies, all of which are extensions of the studies included in this meta-analysis (Loos et al. 2011; Ketter et al. 2016; Pikalov et al. 2017). Two of the three studies evaluated the efficacy and safety of lurasidone therapy adjunctive to baseline Li or VPA therapy, with follow-up periods of 24 weeks and 2 years, respectively. These two studies reported that the efficacy of adjunctive lurasidone therapy was maintained and that the treatment was safe and tolerable during the study periods (Ketter et al. 2016; Pikalov et al. 2017). Another study evaluating the efficacy and safety of adjunctive LTG therapy to Li monotherapy with a follow-up period of 68 weeks reported that the proportion of responders was larger in the LTG group than in the placebo group throughout the study period (Loos et al. 2011). In addition, this study also reported no differences in the prevalence of any adverse events and occurrence of severe adverse events between LTG and a placebo (Loos et al. 2011). Although the results of these studies support the efficacy and safety of adjunctive therapy for treatment-resistant bipolar depression, the overall long-term efficacy and safety of adjunctive SGA or MS therapy are unclear. Therefore, it might be desirable to use adjunctive combination therapy in the short term if possible.

Our subgroup analysis showed small heterogeneities in remission rates from depressive episodes, in the improvement of depressive symptoms, in dropout rates, and in manic switch rates between SGA + MS vs MS + MS. However, there was only one study on MS + MS therapy, which would be insufficient to investigate heterogeneity. Future controlled trials are warranted to confirm the efficacy and safety of adjunctive combination therapies for bipolar depression with inadequate response to monotherapy.

This study has several limitations. First, we included only five RCTs with a total of 1328 participants, resulting in relatively low statistical power. Therefore, further RCTs with larger sample sizes are needed to clarify the effectiveness of SGA or MS adjunctive therapy to SGA or MS monotherapy in bipolar depression. Second, the intervention and baseline treatments differed across the studies included in our meta-analysis, which may have led to heterogeneity of individual interventions in our results. In addition, the baseline treatment in the included studies was only MS monotherapy, although quetiapine and lurasidone monotherapy is recommended as the first-line treatment for bipolar depression by many treatment guidelines. Third, there is a difference between the setting of this study and clinical setting in the real world. Although this study showed that adjunctive therapy was more effective than monotherapy for bipolar depression, there were a lot of patients who already took polypharmacy (Frye et al. 2000). One study reported that the patients with bipolar disorder are treated naturalistically with a mean of 4.1 psychotropic medications during the year (Post et al. 2003). Furthermore, the percentage of monotherapy has decreased from 67 to 31% in the first year of treatment for bipolar disorder (Baldessarini et al. 2008). Therefore, it is difficult to interpret these results into a real world clinical setting. Fourth, there were problems in combining the different pharmacological interventions, SGA + MS and MS + MS, into one analysis. We attempted a meta-analysis with every SGA/MS combination, resulting in fewer interventions and increased heterogeneity. Finally, this study included only short-term RCTs with a study duration of 6 or 8 weeks. Therefore, the long-term efficacy and safety of adjunctive therapy for bipolar depression remain unclear. Further studies are needed to comprehensively investigate the efficacy and safety of adjunctive SGA or MS therapy to SGA or MS monotherapy in bipolar depression.

## Conclusions

Adjunctive SGA or MS therapy could increase the benefits and risks in bipolar depression with inadequate response to LTG, Li, or VPA monotherapy. However, long-term efficacy and safety are unclear. Therefore, adjunctive combination therapy should be provided through shared decision-making, while considering the comorbidities and conditions of patients in clinical settings.

## Supplementary Information

**Additional file 1: Table S1.** PRISMA checklist.

**Additional file 2: Table S2.** Search strategies. **Table S3.** List of the excluded articles.

**Additional file 3: Table S4.** Details of permitted concomitant medications.

## Data Availability

Derived from the original published epidemiological studies.

## Abbreviations

CENTRAL: Cochrane Central Register of Controlled Trials
CI: Confidence interval
CINP: The International College of Neuropsychopharmacology
ECT: Electroconvulsive therapy
HDRS-21: 21-Item Hamilton Depression Rating Scale
Li: Lithium
LTG: Lamotrigine
MADRS: Montgomery Åsberg Depression Rating Scale
MS: Mood stabilizers
QOL: Quality of life
RCTs: Randomized controlled trials
RR: Relative risk
SGA: Second-generation antipsychotic drugs
SMD: Standardized mean differences
VPA: Valproic acid
YMRS: Young Mania Rating Scale
