# Supplementary material for: Efficacy and safety of adjunctive therapy to lamotrigine, lithium, or valproate monotherapy in bipolar depression: a systematic review and meta-analysis of randomized controlled trials
Source: Int J Bipolar Disord. 2022 Oct 21;10:24. doi: 10.1186/s40345-022-00271-7 (PMC9587199; doi:10.1186/s40345-022-00271-7)
Supplement: Supplementary file 2 — Additional file 2: Table S2. Search strategies. Table S3. List of the excluded articles. [file 40345_2022_271_MOESM2_ESM.docx]

**Additional file 2**

**Table S2. Search strategies**

We searched the databases of PubMed, CENTRAL, and Embase using the following term:

PubMed

1. "bipolar disorder"[MeSH Terms] OR ("bipolar"[All Fields] AND "disorder"[All Fields]) OR "bipolar disorder"[All Fields] OR ("bipolar"[All Fields] AND "depression"[All Fields]) OR "bipolar depression"[All Fields]
2. "acepromazine"[MeSH Terms] OR "acepromazine"[All Fields] OR ("acetophenazine"[Supplementary Concept] OR "acetophenazine"[All Fields]) OR ("benperidol"[MeSH Terms] OR "benperidol"[All Fields]) OR ("bromperidol"[Supplementary Concept] OR "bromperidol"[All Fields]) OR ("butaperazine"[Supplementary Concept] OR "butaperazine"[All Fields]) OR ("chlorproethazine"[Supplementary Concept] OR "chlorproethazine"[All Fields]) OR ("chlorpromazin"[All Fields] OR "chlorpromazine"[MeSH Terms] OR "chlorpromazine"[All Fields] OR "chlorpromazine s"[All Fields] OR "chlorpromazines"[All Fields]) OR ("chlorprothixene"[MeSH Terms] OR "chlorprothixene"[All Fields] OR "chlorprothixen"[All Fields]) OR ("clopenthixol"[MeSH Terms] OR "clopenthixol"[All Fields]) OR ("cyamemazine"[Supplementary Concept] OR "cyamemazine"[All Fields]) OR ("dixyrazine"[Supplementary Concept] OR "dixyrazine"[All Fields]) OR ("droperidol"[MeSH Terms] OR "droperidol"[All Fields]) OR ("fluanisone"[Supplementary Concept] OR "fluanisone"[All Fields]) OR ("flupenthixol"[MeSH Terms] OR "flupenthixol"[All Fields] OR "flupentixol"[All Fields]) OR ("fluphenazine"[MeSH Terms] OR "fluphenazine"[All Fields]) OR ("fluspirilene"[MeSH Terms] OR "fluspirilene"[All Fields]) OR ("haloperidol"[MeSH Terms] OR "haloperidol"[All Fields] OR "haloperidol s"[All Fields] OR "haloperidole"[All Fields]) OR ("methotrimeprazine"[MeSH Terms] OR "methotrimeprazine"[All Fields] OR "levomepromazine"[All Fields]) OR ("lenperone"[Supplementary Concept] OR "lenperone"[All Fields]) OR ("loxapine"[MeSH Terms] OR "loxapine"[All Fields]) OR ("mesoridazine"[MeSH Terms] OR "mesoridazine"[All Fields]) OR ("methiothepin"[MeSH Terms] OR "methiothepin"[All Fields] OR "metitepine"[All Fields]) OR ("molindone"[MeSH Terms] OR "molindone"[All Fields]) OR ("moperone"[Supplementary Concept] OR "moperone"[All Fields]) OR ("oxypertine"[Supplementary Concept] OR "oxypertine"[All Fields]) OR ("penfluridol"[MeSH Terms] OR "penfluridol"[All Fields]) OR ("perazin"[All Fields] OR "perazine"[MeSH Terms] OR "perazine"[All Fields]) OR ("periciazine"[Supplementary Concept] OR "periciazine"[All Fields]) OR ("perphenazine"[MeSH Terms] OR "perphenazine"[All Fields]) OR ("pimozide"[MeSH Terms] OR "pimozide"[All Fields]) OR ("pipamperon"[All Fields] OR "pipamperone"[Supplementary Concept] OR "pipamperone"[All Fields]) OR ("piperacetazine"[Supplementary Concept] OR "piperacetazine"[All Fields]) OR ("pipothiazine"[Supplementary Concept] OR "pipothiazine"[All Fields] OR "pipotiazine"[All Fields]) OR ("prochlorperazine"[MeSH Terms] OR "prochlorperazine"[All Fields]) OR ("promazine"[MeSH Terms] OR "promazine"[All Fields] OR "promazines"[All Fields]) OR ("prothipendyl"[Supplementary Concept] OR "prothipendyl"[All Fields]) OR ("spiperone"[MeSH Terms] OR "spiperone"[All Fields]) OR ("sulforidazine"[Supplementary Concept] OR "sulforidazine"[All Fields]) OR ("thiopropazate"[Supplementary Concept] OR "thiopropazate"[All Fields]) OR ("thioproperazine"[Supplementary Concept] OR "thioproperazine"[All Fields]) OR ("thioridazine"[MeSH Terms] OR "thioridazine"[All Fields]) OR ("thiothixene"[MeSH Terms] OR "thiothixene"[All Fields] OR "tiotixene"[All Fields]) OR ("timiperone"[Supplementary Concept] OR "timiperone"[All Fields]) OR ("trifluoperazine"[MeSH Terms] OR "trifluoperazine"[All Fields]) OR ("trifluperidol"[MeSH Terms] OR "trifluperidol"[All Fields]) OR ("triflupromazine"[MeSH Terms] OR "triflupromazine"[All Fields]) OR ("clopenthixol"[MeSH Terms] OR "clopenthixol"[All Fields] OR "zuclopenthixol"[All Fields]) OR ("amoxapine"[MeSH Terms] OR "amoxapine"[All Fields]) OR ("amisulpride"[MeSH Terms] OR "amisulpride"[All Fields]) OR ("aripiprazole"[MeSH Terms] OR "aripiprazole"[All Fields] OR "aripiprazol"[All Fields] OR "aripiprazole s"[All Fields]) OR ("asenapine"[Supplementary Concept] OR "asenapine"[All Fields] OR "asenapine"[All Fields] OR "asenapine s"[All Fields]) OR ("blonanserin"[Supplementary Concept] OR "blonanserin"[All Fields]) OR ("brexpiprazole"[Supplementary Concept] OR "brexpiprazole"[All Fields]) OR ("cariprazine"[Supplementary Concept] OR "cariprazine"[All Fields]) OR ("carpipramine"[Supplementary Concept] OR "carpipramine"[All Fields]) OR ("3 chlorocarpipramine"[Supplementary Concept] OR "3 chlorocarpipramine"[All Fields] OR "clocapramine"[All Fields]) OR ("octoclothepine"[Supplementary Concept] OR "octoclothepine"[All Fields] OR "clorotepine"[All Fields]) OR ("clothiapine"[Supplementary Concept] OR "clothiapine"[All Fields] OR "clotiapine"[All Fields]) OR ("clozapine"[MeSH Terms] OR "clozapine"[All Fields] OR "clozapin"[All Fields] OR "clozapine s"[All Fields]) OR ("iloperidone"[Supplementary Concept] OR "iloperidone"[All Fields]) OR ("levosulpiride"[Supplementary Concept] OR "levosulpiride"[All Fields]) OR ("lurasidone hydrochloride"[MeSH Terms] OR ("lurasidone"[All Fields] AND "hydrochloride"[All Fields]) OR "lurasidone hydrochloride"[All Fields] OR "lurasidone"[All Fields] OR "lurasidone s"[All Fields]) OR ("metylperon"[Supplementary Concept] OR "metylperon"[All Fields] OR "melperon"[All Fields] OR "melperone"[All Fields]) OR ("mosapramine"[Supplementary Concept] OR "mosapramine"[All Fields]) OR ("nemonapride"[Supplementary Concept] OR "nemonapride"[All Fields]) OR ("olanzapine"[MeSH Terms] OR "olanzapine"[All Fields] OR "olanzapin"[All Fields] OR "olanzapine s"[All Fields]) OR ("paliperidone palmitate"[MeSH Terms] OR ("paliperidone"[All Fields] AND "palmitate"[All Fields]) OR "paliperidone palmitate"[All Fields] OR "paliperidone"[All Fields] OR "paliperidone s"[All Fields]) OR ("perospirone"[Supplementary Concept] OR "perospirone"[All Fields]) OR ("quetiapin"[All Fields] OR "quetiapine fumarate"[MeSH Terms] OR ("quetiapine"[All Fields] AND "fumarate"[All Fields]) OR "quetiapine fumarate"[All Fields] OR "quetiapine"[All Fields] OR "quetiapine s"[All Fields]) OR ("remoxipride"[MeSH Terms] OR "remoxipride"[All Fields]) OR ("reserpine"[MeSH Terms] OR "reserpine"[All Fields] OR "reserpin"[All Fields] OR "reserpine s"[All Fields] OR "reserpinization"[All Fields] OR "reserpinized"[All Fields]) OR ("risperidon"[All Fields] OR "risperidone"[MeSH Terms] OR "risperidone"[All Fields] OR "risperidone s"[All Fields]) OR ("sertindol"[All Fields] OR "sertindole"[Supplementary Concept] OR "sertindole"[All Fields]) OR ("sulpiride"[MeSH Terms] OR "sulpiride"[All Fields] OR "sulpirid"[All Fields]) OR ("amisulpride"[MeSH Terms] OR "amisulpride"[All Fields] OR "sultopride"[All Fields]) OR ("tiaprid"[All Fields] OR "tiapride hydrochloride"[MeSH Terms] OR ("tiapride"[All Fields] AND "hydrochloride"[All Fields]) OR "tiapride hydrochloride"[All Fields] OR "tiapridal"[All Fields] OR "tiapride"[All Fields]) OR ("veralipride"[Supplementary Concept] OR "veralipride"[All Fields]) OR ("ziprasidone"[Supplementary Concept] OR "ziprasidone"[All Fields] OR "ziprasidone s"[All Fields]) OR ("zotepine"[Supplementary Concept] OR "zotepine"[All Fields] OR "zotepine s"[All Fields])
3. "lithium"[MeSH Terms] OR "lithium"[All Fields] OR "lithium s"[All Fields] OR "lithiums"[All Fields]
4. "valproic acid"[MeSH Terms] OR ("valproic"[All Fields] AND "acid"[All Fields]) OR "valproic acid"[All Fields]
5. "lamotrigin"[All Fields] OR "lamotrigine"[MeSH Terms] OR "lamotrigine"[All Fields] OR "lamotrigine s"[All Fields]
6. "carbamazepine"[MeSH Terms] OR "carbamazepine"[All Fields] OR "carbamazepin"[All Fields] OR "carbamazepines"[All Fields] OR "carbamazepine s"[All Fields]
7. "drug synergism"[MeSH Terms] OR ("drug"[All Fields] AND "synergism"[All Fields]) OR "drug synergism"[All Fields]
8. ("randomized controlled trial"[Publication Type] OR "controlled clinical trial"[Publication Type] OR "randomized"[Title/Abstract] OR "placebo"[Title/Abstract] OR "drug therapy"[MeSH Subheading] OR "randomly"[Title/Abstract] OR "trial"[Title/Abstract]) AND "groups"[Title/Abstract]
9. 2 OR 3 OR 4 OR 5 OR 6 OR 7
10. 1 AND 8 AND 9

CENTRAL

1. MeSH descriptor: [Bipolar Disorder] explode all trees
2. acepromazine OR acetophenazine OR benperidol OR bromperidol OR butaperazine OR carfenazine OR chlorproethazine OR chlorpromazine OR chlorprothixene OR clopenthixol OR cyamemazine OR dixyrazine OR droperidol OR fluanisone OR flupentixol OR fluphenazine OR fluspirilene OR haloperidol OR levomepromazine OR lenperone OR loxapine OR mesoridazine OR metitepine OR molindone OR moperone OR oxypertine OR oxyprotepine OR penfluridol OR perazine OR periciazine OR perphenazine OR pimozide OR pipamperone OR piperacetazine OR pipotiazine OR prochlorperazine OR promazine OR prothipendyl OR spiperone OR sulforidazine OR thiopropazate OR thioproperazine OR thioridazine OR thiothixene OR timiperone OR trifluoperazine OR trifluperidol OR triflupromazine OR zuclopenthixol OR amoxapine OR amisulpride OR aripiprazole OR asenapine OR blonanserin OR brexpiprazole OR cariprazine OR carpipramine OR clocapramine OR clorotepine OR clotiapine OR clozapine OR iloperidone OR levosulpiride OR lurasidone OR melperone OR mosapramine OR nemonapride OR olanzapine OR paliperidone OR perospirone OR quetiapine OR remoxipride OR reserpine OR risperidone OR sertindole OR sulpiride OR sultopride OR tiapride OR veralipride OR ziprasidone OR zotepine
3. MeSH descriptor: [Lithium] explode all trees
4. MeSH descriptor: [Valproic Acid] explode all trees
5. 'MeSH descriptor: [Lamotrigine] explode all trees
6. MeSH descriptor: [Carbamazepine] explode all trees
7. MeSH descriptor: [Drug Synergism] explode all trees
8. #2 OR #3 OR #4 OR #5 OR #6 OR #7
9. #1 AND #8

Embase

1. 'bipolar depression'
2. acepromazine OR acetophenazine OR benperidol OR bromperidol OR butaperazine OR carfenazine OR chlorproethazine OR chlorpromazine OR chlorprothixene OR clopenthixol OR cyamemazine OR dixyrazine OR droperidol OR fluanisone OR flupentixol OR fluphenazine OR fluspirilene OR haloperidol OR levomepromazine OR lenperone OR loxapine OR mesoridazine OR metitepine OR molindone OR moperone OR oxypertine OR oxyprotepine OR penfluridol OR perazine OR periciazine OR perphenazine OR pimozide OR pipamperone OR piperacetazine OR pipotiazine OR prochlorperazine OR promazine OR prothipendyl OR spiperone OR sulforidazine OR thiopropazate OR thioproperazine OR thioridazine OR thiothixene OR timiperone OR trifluoperazine OR trifluperidol OR triflupromazine OR zuclopenthixol OR amoxapine OR amisulpride OR aripiprazole OR asenapine OR blonanserin OR brexpiprazole OR cariprazine OR carpipramine OR clocapramine OR clorotepine OR clotiapine OR clozapine OR iloperidone OR levosulpiride OR lurasidone OR melperone OR mosapramine OR nemonapride OR olanzapine OR paliperidone OR perospirone OR quetiapine OR remoxipride OR reserpine OR risperidone OR sertindole OR sulpiride OR sultopride OR tiapride OR veralipride OR ziprasidone OR zotepine
3. lithium
4. 'valproic acid'
5. lamotrigine
6. carbamazepine
7. 'drug potentiation'
8. 'crossover procedure':de OR 'double-blind procedure':de OR 'randomized controlled trial':de OR 'single-blind procedure':de OR random*:de,ab,ti OR factorial*:de,ab,ti OR crossover*:de,ab,ti OR ((cross NEXT/1 over*):de,ab,ti) OR placebo*:de,ab,ti OR ((doubl* NEAR/1 blind*):de,ab,ti) OR ((singl* NEAR/1 blind*):de,ab,ti) OR assign*:de,ab,ti OR allocat*:de,ab,ti OR volunteer*:de,ab,ti
9. 2 OR 3 OR 4 OR 5 OR 6 OR 7
10. 1 AND 9
11. 8 AND 10

**Table S3. List of the excluded articles**

| Study | Reason for exclusion |
| --- | --- |
| Brown 2014 ^1^ | not adequate for the patients |
| Brown 2014 ^2^ | Duplicated |
| Brown 2008 ^3^ | not adequate for the patients |
| Calabrese 2014 ^4^ | not identifying full text, Conference article |
| Calabrese2015 ^5^ | not identifying full text, Conference article |
| Calabrese2013 ^6^ | not identifying full text, Conference article |
| Chapel 2015 ^7^ | not identifying full text, Conference article |
| Chapel 2015 ^8^ | not identifying full text, Conference article |
| Chapel 2016 ^9^ | Not adequate for the outcomes |
| Citrome 2013 ^10^ | Relevant to other article, Conference article |
| Citrome 2014 ^11^ | Relevant to other article, Conference article |
| Dixit 2014 ^12^ | Relevant to other article, Conference article |
| Forester 2018 ^13^ | Not adequate for the control and trial design |
| Forester 2015 ^14^ | Relevant to other article, Conference article |
| Forester 2015 ^15^ | Relevant to other article, Conference article |
| Forester 2015 ^16^ | Relevant to other article, Conference article |
| Frye 2015 ^17^ | Relevant to other article, Conference article |
| Gao 2013 ^18^ | Relevant to other article, Conference article |
| Gao 2014 ^19^ | Not adequate for the patients |
| Gao 2014 ^20^ | Duplicate |
| Gardiner 2011 ^21^ | Relevant to other article, Conference article |
| Garriga 2017 ^22^ | Not adequate for the patients |
| Geddes 2014 ^23^ | Relevant to other article, Conference article |
| Geddes 2016 ^24^ | Not adequate for the patients |
| Geddes 2016 ^25^ | Duplicate |
| Geddes 2016 ^26^ | Duplicate |
| Geddes 2015 ^27^ | Relevant to other article, Conference article |
| Geddes 2015 ^28^ | Relevant to other article, Conference article |
| Hirschfeld 2012 ^29^ | Relevant to other article, Conference article |
| Houston 2010 ^30^ | Not adequate for the patients and outcomes |
| Houston 2011 ^31^ | Not adequate for the patients |
| Iosifescu 2014 ^32^ | Relevant to other article, Conference article |
| Iosifescu 2015 ^33^ | Relevant to other article, Conference article |
| Kemp 2012 ^34^ | Not adequate for the intervention and control |
| Ketter 2013 ^35^ | Relevant to other article, Conference article |
| Ketter 2013 ^36^ | Relevant to other article, Conference article |
| Ketter 2012 ^37^ | Relevant to other article, Conference article |
| Ketter 2015 ^38^ | Relevant to other article, Conference article |
| Ketter 2016 ^39^ | Not adequate for the control and study design |
| Ketter 2015 ^40^ | Relevant to other article, Conference article |
| Loebel 2015 ^41^ | Relevant to other article, Conference article |
| Loebel 2014 ^42^ | Relevant to other article, Conference article |
| McElroy 2014 ^43^ | Relevant to other article, Conference article |
| McElroy 2015 ^44^ | Relevant to other article, Conference article |
| Namjoshi 2004 ^45^ | Not adequate for the patient |
| NCT00000439 ^46^ | Not adequate for the patient, protocol article |
| NCT00063362 ^47^ | Relevant to other article, protocol article |
| NCT00183469 ^48^ | Not adequate for the patient, protocol article |
| NCT00224510 ^49^ | Relevant to other article, protocol article |
| NCT00240110 ^50^ | Not adequate for the trial design, protocol article |
| NCT00483548 ^51^ | Relevant to other article, protocol article |
| NCT00671853^5 2^ | Not adequate for the patient, protocol article |
| NCT01015586 ^53^ | Not adequate for the trial design, protocol article |
| NCT00835107 ^54^ | Not adequate for the intervention and control, protocol article |
| NCT00868452 ^55^ | Relevant to other article, protocol article |
| NCT01211704 ^56^ | not identifying full text, protocol article |
| NCT01284517 ^57^ | Relevant to other article, protocol article |
| NCT01588457 ^58^ | Not adequate for the patient, protocol article |
| NCT01938859 ^59^ | Not adequate for the trial design, protocol article |
| NCT03423680 ^60^ | not identifying full text, protocol article |
| Newcomer 2014 ^61^ | Relevant to other article, Conference article |
| Newcomer 2015 ^62^ | Relevant to other article, Conference article |
| Newcomer 2015 ^63^ | Relevant to other article, Conference article |
| Pae 2012 ^64^ | Not adequate for the patient |
| Pikalov 2016 ^65^ | Relevant to other article, Conference article |
| Pikalov 2016 ^66^ | Not adequate for the patient, Conference article |
| Pikalov 2015 ^67^ | Not adequate for the trial design, Conference article |
| Pikalov 2017 ^68^ | Not identifying full text, Conference article |
| Pikalov 2016 ^69^ | Relevant to other article, Conference article |
| Quante 2010 ^70^ | Not adequate for trial design |
| Ravindran 2004 ^71^ | Not adequate for the patient |
| Sajatovic 2014 ^72^ | Relevant to other article, Conference article |
| Sajatovic 2015 ^73^ | Relevant to other article, Conference article |
| Sajatovic 2015 ^74^ | Relevant to other article, Conference article |
| Sajatovic 2015 ^75^ | Relevant to other article, Conference article |
| Sajatovic 2016 ^76^ | Relevant to other article, Conference article |
| Sajatovic 2015 ^77^ | Relevant to other article, Conference article |
| Silva Lima 2019 ^78^ | Not adequate for trial design |
| Simon 2018 ^79^ | Relevant to other article, Conference article |
| Solomon 1997 ^80^ | Not adequate for the patient |
| Stedman 2010 ^81^ | Not adequate for the patient |
| Su 2011 ^82^ | not identifying full text |
| Tocco 2020 ^83^ | not identifying full text, Conference article |
| Tocco 2020 ^84^ | Relevant to other article, Conference article |
| Van Der Loos 2011 ^85^ | Duplicate |
| Van Der Loos 2010 ^86^ | Duplicate |
| Van Der Loos 2009 ^87^ | Duplicate |

1. Brown ES, Davila D, Nakamura A, et al. A randomized, double-blind, placebo-controlled trial of quetiapine in patients with bipolar disorder, mixed or depressed phase, and alcohol dependence. *Alcoholism, clinical and experimental research.* 2014;38(7):2113‐2118.

2. Brown ES, Davila D, Nakamura A, et al. A randomized, double-blind, placebo-controlled trial of quetiapine in patients with bipolar disorder, mixed or depressed phase, and alcohol dependence. *Alcohol Clin Exp Res.*38(7):2113-2118.

3. Brown ES, Garza M, Carmody TJ. A randomized, double-blind, placebo-controlled add-on trial of quetiapine in outpatients with bipolar disorder and alcohol use disorders. *Journal of clinical psychiatry.* 2008;69(5):701‐705.

4. Calabrese J, Suppes T, Sarma K, et al. Efficacy and safety of treatment with lurasidone adjunctive with lithium or valproate in bipolar I depression: Results of two 6-week studies. *International Journal of Neuropsychopharmacology.* 2014;17:52.

5. Calabrese J, Suppes T, Sarma K, et al. Efficacy and safety of treatment with lurasidone adjunctive with lithium or valproate in bipolar i depression: Results of two 6-week studies. *CNS spectrums.* 2015;20(1):72.

6. Calabrese JR, Ketter TA, Cucchiaro J, et al. A post hoc analysis of efficacy and tolerability of lurasidone adjunctive to either lithium or valproate for the treatment of bipolar I depression. *Bipolar disorders.* 2013;15:131-132.

7. Chapel S, Chiu YY, Hsu J, Cucchiaro J, Loebel A. Efficacy of lurasidone in bipolar depression: Population exposure-response relationships in patients with bipolar depression. *Neuropsychopharmacology : official publication of the American College of Neuropsychopharmacology.* 2015;40:S140-S141.

8. Chapel S, Chiu YY, Hsu J, Cucchiaro J, Loebel A. Exposure-response model of lurasidone in patients with bipolar depression. *CNS spectrums.* 2015;20(1):70-71.

9. Chapel S, Chiu YY, Hsu J, Cucchiaro J, Loebel A. Lurasidone Dose Response in Bipolar Depression: A Population Dose-response Analysis. *Clinical Therapeutics.* 2016;38(1):4-15.

10. Citrome L, Ketter TA, Cucchiaro J, Loebel A. Clinical assessment of lurasidone benefit and risk in the treatment of bipolar I depression using number needed to treat, number needed to harm, and likelihood to be helped or harmed. *Neuropsychopharmacology : official publication of the American College of Neuropsychopharmacology.* 2013;38:S359-S361.

11. Citrome L, Ketter T, Cucchiaro J, Loebel A. Clinical assessment of lurasidone benefit and risk in the treatment of bipolar I depression using number needed to treat, number needed to harm, and likelihood to be helped or harmed. *International Journal of Neuropsychopharmacology.* 2014;17:54.

12. Dixit M, Dhavale D. Lurasidone - A double blind trial in bipolar depression. *Indian Journal of Psychiatry.* 2014;56:S73.

13. Forester BP, Sajatovic M, Tsai J, Pikalov A, Cucchiaro J, Loebel A. Safety and Effectiveness of Long-Term Treatment with Lurasidone in Older Adults with Bipolar Depression: Post-Hoc Analysis of a 6-Month, Open-Label Study. *American Journal of Geriatric Psychiatry.* 2018;26(2):150-159.

14. Forester B, Sajatovic M, Tsai J, et al. Long-term treatment with lurasidone in older adults with bipolar depression: Results of a 6 month open-label study. *European Psychiatry.* 2015;30:1134.

15. Forester B, Sajatovic M, Tsai J, et al. Efficacy and safety of long-term treatment with lurasidone in older adults with bipolar depression: Results of a 6 month open-label study. *American Journal of Geriatric Psychiatry.* 2015;23(3):S170-S171.

16. Forester B, Sajatovic M, Tsai J, et al. Efficacy and safety of long-term treatment with lurasidone in older adults with bipolar depression: Results of a 6 month open-label study. *Bipolar disorders.* 2015;17:132.

17. Frye M, Tsai J, Kroger H, Pikalov A, Cucchiaro J, Loebel A. Lurasidone in bipolar depression studies: Dose utilization frequencies. *Bipolar disorders.* 2015;17:133.

18. Gao K, Kemp D, Conroy C, et al. Efficacy and safety of quetiapine-XR monotherapy or adjunctive therapy to mood stabilizer in the treatment of comorbid generalized anxiety disorder in bipolar depression with or without substance use disorder. *Bipolar disorders.* 2013;15:59-60.

19. Gao K, Wu R, Kemp DE, et al. Efficacy and safety of quetiapine-XR as monotherapy or adjunctive therapy to a mood stabilizer in acute bipolar depression with generalized anxiety disorder and other comorbidities: A randomized, placebo-controlled trial. *Journal of Clinical Psychiatry.* 2014;75(10):1062-1068.

20. Gao K, Wu R, Kemp DE, et al. Efficacy and safety of quetiapine-XR as monotherapy or adjunctive therapy to a mood stabilizer in acute bipolar depression with generalized anxiety disorder and other comorbidities: a randomized, placebo-controlled trial. *Journal of clinical psychiatry.* 2014;75(10):1062‐1068.

21. Gardiner A, Rendell J, Stephens W, et al. CEQUEL: A Comparative Evaluation of QUEtiapine-Lamotrigine combination versus quetiapine monotherapy, (and folic acid versus placebo) in people with bipolar depression: A 2x2 factorial randomised trial. *Bipolar disorders.* 2011;13:21-22.

22. Garriga M, Solé E, González-Pinto A, et al. Efficacy of quetiapine XR vs. placebo as concomitant treatment to mood stabilizers in the control of subthreshold symptoms of bipolar disorder: Results from a pilot, randomized controlled trial. *European neuropsychopharmacology : the journal of the European College of Neuropsychopharmacology.*27(10):959-969.

23. Geddes J, Hinds C, Rendell J, et al. Comparative evaluation of quetiapine plus lamotrigine versus quetiapine monotherapy in bipolar depression: A randomized placebo controlled trial (CEQUEL). *Neuropsychopharmacology : official publication of the American College of Neuropsychopharmacology.* 2014;39:S371.

24. Geddes JR, Gardiner A, Rendell J, et al. Comparative evaluation of quetiapine plus lamotrigine combination versus quetiapine monotherapy (and folic acid versus placebo) in bipolar depression (CEQUEL): a 2 × 2 factorial randomised trial. *The lancet Psychiatry.* 2016;3(1):31‐39.

25. Geddes JR, Gardiner A, Rendell J, et al. Comparative evaluation of quetiapine plus lamotrigine combination versus quetiapine monotherapy (and folic acid versus placebo) in bipolar depression (CEQUEL): a 2 × 2 factorial randomised trial. *The lancet Psychiatry.*3(1):31-39.

26. Geddes JR, Gardiner A, Rendell J, et al. Comparative evaluation of quetiapine plus lamotrigine combination versus quetiapine monotherapy (and folic acid versus placebo) in bipolar depression (CEQUEL): A 2 × 2 factorial randomised trial. *The Lancet Psychiatry.* 2016;3(1):31-39.

27. Geddes JR, Hinds C, Rendell J, et al. Comparative evaluation of quetiapine plus lamotrigine versus quetiapine monotherapy in people with bipolar depression: A randomized trial (CEQUEL). *Bipolar disorders.* 2015;17:25.

28. Geddes JR, Rendell J, Hinds C, et al. Comparative evaluation of quetiapine plus lamotrigine versus quatiapine monotherapy in people with bipolar depression: A randomized trial (CEQUEL). *Bipolar disorders.* 2015;17:50-51.

29. Hirschfeld RMA, Cucchiaro J, Pikalov A, et al. Effect of lurasidone monotherapy or adjunctive therapy on anxiety symptoms in patients with bipolar I depression. *Neuropsychopharmacology : official publication of the American College of Neuropsychopharmacology.* 2012;38:S423-S424.

30. Houston JP, Gatz JL, Degenhardt EK, Jamal HH. Symptoms predicting remission after divalproex augmentation with olanzapine in partially nonresponsive patients experiencing mixed bipolar I episode: a post-hoc analysis of a randomized controlled study. *BMC Res Notes.*3:276.

31. Houston JP, Ketter TA, Case M, et al. Early symptom change and prediction of subsequent remission with olanzapine augmentation in divalproex-resistant bipolar mixed episodes. *Journal of psychiatric research.* 2011;45(2):169‐173.

32. Iosifescu D, Tsai J, Pikalov A, Hsu J, Cucchiaro J, Loebel A. Lurasidone in bipolar disorder: Early improvement as a predictor of short-term response. *Neuropsychopharmacology : official publication of the American College of Neuropsychopharmacology.* 2014;39:S380-S381.

33. Iosifescu DV, Tsai J, Pikalov A, Kroger H, Cucchiaro J, Loebel A. The value of early improvement as a predictor of short-term response during treatment of bipolar depression with lurasidone. *European Neuropsychopharmacology.* 2015;25:S421.

34. Kemp DE, Gao K, Fein EB, et al. Lamotrigine as add-on treatment to lithium and divalproex: Lessons learned from a double-blind, placebo-controlled trial in rapid-cycling bipolar disorder. *Bipolar disorders.* 2012;14(7):780-789.

35. Ketter TA, Citrome L, Cucchiaro J, Loebel A. Clinical assessment of lurasidone benefit and risk in the treatment of bipolar I depression using number needed to treat, number needed to harm, and likelihood to be helped or harmed. *Bipolar disorders.* 2013;15:92.

36. Ketter TA, Cucchiaro J, Silva R, et al. Lurasidone treatment for bipolar I depression: Effects on quality of life and patient's functioning. *Bipolar disorders.* 2013;15:91.

37. Ketter TA, Cucchiaro J, Silva R, et al. Lurasidone for bipolar i depression: Effects on quality of life and functioning. *Neuropsychopharmacology : official publication of the American College of Neuropsychopharmacology.* 2012;38:S169-S170.

38. Ketter T, Tsai J, Silva R, Kroger H, Cucchiaro J, Loebel A. Lurasidone in the long-term treatment of patients with bipolar i disorder: Responder and remitter status during a 24-week open-label extension study. *European Neuropsychopharmacology.* 2015;25:S420-S421.

39. Ketter TA, Sarma K, Silva R, Kroger H, Cucchiaro J, Loebel A. LURASIDONE in the LONG-TERM TREATMENT of PATIENTS with BIPOLAR DISORDER: A 24-WEEK OPEN-LABEL EXTENSION STUDY. *Depression and Anxiety.* 2016;33(5):424-434.

40. Ketter TA, Sarma K, Silva R, Kroger H, Cucchiaro J, Loebel A. Lurasidone in bipolar i depression: A 24 week, open-label extension study. *CNS spectrums.* 2015;20(1):72-73.

41. Loebel A, Cucchiaro J, Kroger H, Pikalov A. Lurasidone for the treatment of bipolar depression: Current state of the evidence. *CNS spectrums.* 2015;20(1):74-75.

42. Loebel A, Cucchiaro J, Silva R, et al. Efficacy and safety of lurasidone in bipolar depression: Results from two, double blind, placebo-controlled studies. *Bipolar disorders.* 2014;16:131-132.

43. McElroy S, Pikalov A, Cucchiaro J, et al. Short- and longer-term treatment with lurasidone in patients with bipolar I depression: Effect on metabolic syndrome. *International Journal of Neuropsychopharmacology.* 2014;17:93.

44. McElroy S, Pikalov A, Cucchiaro J, et al. Short-and longer-term treatment with lurasidone in patients with bipolar i depression: Effect on metabolic syndrome. *CNS spectrums.* 2015;20(1):71-72.

45. Namjoshi MA, Risser R, Shi L, Tohen M, Breier A. Quality of life assessment in patients with bipolar disorder treated with olanzapine added to lithium or valproic acid. *Journal of affective disorders.* 2004;81(3):223‐229.

46. Nct. Drug Treatment for Alcoholics With Bipolar Disorder. [*https://clinicaltrialsgov/show/NCT00000439*](https://clinicaltrialsgov/show/NCT00000439)*.* 1999.

47. Nct. Combination Therapy for the Treatment of Bipolar Disorders. [*https://clinicaltrialsgov/show/NCT00063362*](https://clinicaltrialsgov/show/NCT00063362)*.* 2003.

48. Nct. Maintenance Treatment of Bipolar Depression. [*https://clinicaltrialsgov/show/NCT00183469*](https://clinicaltrialsgov/show/NCT00183469)*.* 2005.

49. Nct. A Study To Investigate The Antidepressant Effect Of Lamotrigine In Patients With Bipolar Disorder Using Lithium. [*https://clinicaltrialsgov/show/NCT00224510*](https://clinicaltrialsgov/show/NCT00224510)*.* 2005.

50. Nct. Valproate Efficacy in Cocaine-Bipolar Comorbidity. [*https://clinicaltrialsgov/show/NCT00240110*](https://clinicaltrialsgov/show/NCT00240110)*.* 2005.

51. Nct. Adjunctive Ziprasidone in the Treatment of Bipolar I Depression. [*https://clinicaltrialsgov/show/NCT00483548*](https://clinicaltrialsgov/show/NCT00483548)*.* 2007.

52. Nct. Quetiapine Extended Release (XR) in Bipolar Patients With Comorbid Generalized Anxiety Disorder (GAD). [*https://clinicaltrialsgov/show/NCT00671853*](https://clinicaltrialsgov/show/NCT00671853)*.* 2008.

53. Nct. Treatment of Alcohol Dependence and Comorbid Bipolar Disorder. [*https://clinicaltrialsgov/show/NCT01015586*](https://clinicaltrialsgov/show/NCT01015586)*.* 2009.

54. Nct. An Investigation of Sleep Architecture in Ziprasidone-Treated Bipolar Depression. [*https://clinicaltrialsgov/show/NCT00835107*](https://clinicaltrialsgov/show/NCT00835107)*.* 2009.

55. Nct. Lurasidone - A 6-week Study of Patients With Bipolar I Depression (Add-on). [*https://clinicaltrialsgov/show/NCT00868452*](https://clinicaltrialsgov/show/NCT00868452)*.* 2009.

56. Nct. Paliperidone Palmitate Efficacy and Safety in Bipolar Disorder Complicated by Alcoholism. [*https://clinicaltrialsgov/show/NCT01211704*](https://clinicaltrialsgov/show/NCT01211704)*.* 2010.

57. Nct. Lurasidone HCI - A 6-week Phase 3 Study of Patients With Bipolar I Depression. [*https://clinicaltrialsgov/show/NCT01284517*](https://clinicaltrialsgov/show/NCT01284517)*.* 2011.

58. Nct. Sequential Multiple Assignment Treatment for Bipolar Disorder. [*https://clinicaltrialsgov/show/NCT01588457*](https://clinicaltrialsgov/show/NCT01588457)*.* 2012.

59. Nct. Algorithm Guided Treatment Strategies for Bipolar Depression. [*https://clinicaltrialsgov/show/NCT01938859*](https://clinicaltrialsgov/show/NCT01938859)*.* 2013.

60. Nct. A Study of Abilify® Tablet(Aripiprazole) as an Adjunctive Treatment in the Bipolar Depression. [*https://clinicaltrialsgov/show/NCT03423680*](https://clinicaltrialsgov/show/NCT03423680)*.* 2018.

61. Newcomer J, Tsai J, Pikalov A, Kroger H, Cucchiaro J, Loebel A. Effect of lurasidone on metabolic parameters in patients with bipolar depression. *Neuropsychopharmacology : official publication of the American College of Neuropsychopharmacology.* 2014;39:S386.

62. Newcomer JW, Tsai J, Pikalov A, Kroger H, Cucchiaro J. Effect of lurasidone on metabolic parameters in patients with bipolar depression. *European Psychiatry.* 2015;30:1140.

63. Newcomer JW, Tsai J, Pikalov A, Kroger H, Cucchiaro J, Loebel A. Effect of lurasidone on metabolic parameters in patients with bipolar depression. *Bipolar disorders.* 2015;17:88.

64. Pae CU, Mas, PS, el FS, O'Gorman C. Achieving and sustaining remission in bipolar I disorder with ziprasidone : a post hoc analysis of a 24-week, double-blind, placebo-controlled study. *Clin Drug Investig.*32(11):747-754.

65. Pikalov A, Silva R, Cucchiaro J, Sarma K, Hsu J, Loebel A. Efficacy of lurasidone in bipolar depression: Results from two double-blind, placebo-controlled studies. *Australian and New Zealand Journal of Psychiatry.* 2016;50:132.

66. Pikalov A, Tsai J, Cucchiaro J, Loebel A. Lurasidone in the treatment of bipolar depression: Effect of baseline depression severity on clinical outcome. *CNS spectrums.* 2016;21(1):105-106.

67. Pikalov A, Tsai J, Mao Y, Cucchiaro J, Loebel A. Long-term use of lurasidone in patients with bipolar disorder: Safety and effectiveness over 2 years of treatment. *Bipolar disorders.* 2015;17:88-89.

68. Pikalov A, Tsai J, Mao Y, Cucchiaro J, Loebel A. Lurasidone adjunctive to lithium or valproate in patients with bipolar i disorder: Effectiveness of up to 20 weeks of treatment. *European Neuropsychopharmacology.* 2017;27:S825-S826.

69. Pikalov A, Tsai J, Mao Y, Silva R, Cucchiaro J, Loebel A. Long-term use of lurasidone in patients with bipolar disorder: Safety and effectiveness over 2 years of treatment. *CNS spectrums.* 2016;21(1):81.

70. Quante A, Zeugmann S, Luborzewski A, et al. Aripiprazole as adjunct to a mood stabilizer and citalopram in bipolar depression: A randomized placebo-controlled pilot study. *Human psychopharmacology.* 2010;25(2):126-132.

71. Ravindran A, Silverstone P, Lacroix D, et al. Risperidone does not affect steady-state pharmacokinetics of divalproex sodium in patients with bipolar disorder. *Clin Pharmacokinet.* 2004;43(11):733-740.

72. Sajatovic M, Forester B, Tsai J, et al. Efficacy and safety of lurasidone in older adults with bipolar depression: Analysis of two double-blind, placebo-controlled studies. *Neuropsychopharmacology : official publication of the American College of Neuropsychopharmacology.* 2014;39:S348-S349.

73. Sajatovic M, Forester B, Tsai J, et al. Lurasidone for older adults with bipolar depression: Analysis of two double-blind, placebo-controlled studies. *European Neuropsychopharmacology.* 2015;25:S420.

74. Sajatovic M, Forester B, Tsai J, et al. Efficacy and tolerability of lurasidone in older adults with bipolar depression: Analysis of two 6-week double-blind, placebo-controlled studies. *European Psychiatry.* 2015;30:1136.

75. Sajatovic M, Forester B, Tsai J, et al. Efficacy and safety of lurasidone in older adults with bipolar depression: Analysis of two double-blind, placebo-controlled studies. *American Journal of Geriatric Psychiatry.* 2015;23(3):S171.

76. Sajatovic M, Forester B, Tsai J, et al. Efficacy and safety of lurasidone in older adults with bipolar depression. *CNS spectrums.* 2016;21(1):104.

77. Sajatovic M, Foresterb B, Tsai J, et al. Efficacy and safety of lurasidone in older adults with bipolar depression: Analysis of two double-blind, placebo-controlled studies. *Bipolar disorders.* 2015;17:133.

78. Silva Lima AFBD, Cohen M, Miguel S, Cruz L. Comparative economic evaluation of quetiapine plus lamotrigine combination vs quetiapine monotherapy(and folic acid vs placebo) in patients with bipolar depression (CEQUEL). *Bipolar disorders.* 2019;21(2):172-173.

79. Simon J, Geddes JR, Gardiner A, Rendell J, Goodwin GM, Mayer S. Comparative economic evaluation of quetiapine plus lamotrigine combination vs quetiapine monotherapy (and folic acid vs placebo) in patients with bipolar depression (CEQUEL). *Bipolar disorders.* 2018;20(8):733-745.

80. Solomon DA, Ryan CE, Keitner GI, et al. A pilot study of lithium carbonate plus divalproex sodium for the continuation and maintenance treatment of patients with bipolar I disorder. *The Journal of clinical psychiatry.*58(3):95-99.

81. Stedman M, Pettinati HM, Brown ES, Kotz M, Calabrese JR, Raines S. A double-blind, placebo-controlled study with quetiapine as adjunct therapy with lithium or divalproex in bipolar I patients with coexisting alcohol dependence. *Alcohol Clin Exp Res.*34(10):1822-1831.

82. Su YS, Chen J, Li ZZ, et al. Clinical efficacy and safety of lithium carbonate combined with aripiprazole in treatment of bipolar depression. *Journal of Shanghai Jiaotong University (Medical Science).* 2011;31(11):1536-1539.

83. Tocco M, Newcomer JW, Mao Y, Pikalov A. 160 Lurasidone and Metabolic Syndrome: Results from Short- and Long-Term Clinical Studies in Patients with Bipolar Depression. *CNS spectrums.* 2020;25(2):302-303.

84. Tocco M, Newcomer JW, Mao Y, Pikalov A. Lurasidone and metabolic syndrome: Results from short- and long-term clinical studies in patients with bipolar depression. *CNS spectrums.* 2020;25(2):302-303.

85. Van Der Loos MLM, Mulder P, GthM Hartong E, et al. Long-term outcome of bipolar depressed patients receiving lamotrigine as add-on to lithium with the possibility of the addition of paroxetine in nonresponders: A randomized, placebo-controlled trial with a novel design. *Bipolar disorders.* 2011;13(1):111-117.

86. Van Der Loos MLM, Mulder P, Hartong EGTM, et al. Efficacy and safety of two treatment algorithms in bipolar depression consisting of a combination of lithium, lamotrigine or placebo and paroxetine. *Acta psychiatrica Scandinavica.* 2010;122(3):246-254.

87. Van Der Loos MLM, Mulder PGH, Hartong EGTM, et al. Efficacy and safety of lamotrigine as add-on treatment to lithium in bipolar depression: A multicenter, double-blind, placebo-controlled trial. *Journal of Clinical Psychiatry.* 2009;70(2):223-231.
