# Supplementary material for: Efficacy and safety of adjunctive therapy to lamotrigine, lithium, or valproate monotherapy in bipolar depression: a systematic review and meta-analysis of randomized controlled trials
Source: Int J Bipolar Disord. 2022 Oct 21;10:24. doi: 10.1186/s40345-022-00271-7 (PMC9587199; doi:10.1186/s40345-022-00271-7)
Supplement: Supplementary file 3 — Additional file 3: Table S4. Details of permitted concomitant medications. [file 40345_2022_271_MOESM3_ESM.docx]

**Table S4. Details of permitted concomitant medications**

| **Study (year)** | **Intervention/**  **Control** | **Taking benzodiazepine (%)** | **Details of permitted concomitant medications** |
| --- | --- | --- | --- |
| **van der Loos**  **(2009)** | Intervention: 64  LTG | ND | Except for lithium, patients were not allowed to use an antipsychotic or antidepressant within 2 weeks (fluoxetine, 4 weeks) of randomization  Benzodiazepines were allowed at a maximum of 2 mg lorazepam equivalents per day throughout the study, i.e., for standing and rescue medication, for anxiety, agitation, or sleep problems. |
|  | Control: 60  Placebo | ND |  |
| **Houston (2009)** | Intervention: 101  Olanzapine | ND | Concomitant benzodiazepine therapy was permitted for ≤ 15 cumulative days or ≤ 5 consecutive days, with a maximum daily dose of 2 mg of lorazepam or lorazepam equivalents (temazepam 30 mg, diazepam 10 mg, oxazepam 30 mg, or chlordiazepoxide 20 mg) and no more than 1 mg of lorazepam equivalent per single dose. Thyroid hormone supplements for hypothyroidism were permitted only if the participant had been on a stable dose of such medication for at least 2 months prior to visit 3 and had serum thyroid stimulating hormone levels within the normal range at screening. Other concomitant medications with primarily central nervous system activity were not allowed. |
|  | Control: 101  Placebo | ND |  |
| **Sachs**  **(2011)** | Intervention: 148  Ziprasidone | ND | All other psychotropic medications were withdrawn at lowest 7 days or 4 half-lives (whichever was longer) before randomization. Lorazepam, or an alternative short-acting benzodiazepine, could be given at doses of up to 2 mg for up to 4 days per week during screening and the first 2 weeks of the double-blind treatment period to treat agitation or anxiety. Regulatory agency-approved nonbenzodiazepine medications could be used to treat sleep disturbances for up to 4 days per week until the end of the second week of double-blind treatment and for up to 2 days per week thereafter. The benzodiazepines and sleep agents were not to be given on the same day and were not to be used within 24 h of efficacy assessments. Benztropine (≤ 6 mg/d) or an equivalent agent could be used to treat extrapyramidal symptoms. Propranolol (≤ 120 mg/d) could be used to treat akathisia. |
|  | Control: 150  Placebo | ND |  |
| **Loebel**  **(2014)** | Intervention: 183  Lurasidone | ND | Treatment with anticholinergic agents, propranolol, or amantadine was permitted as needed for movement disorders. Lorazepam, temazepam, or zolpidem (or their equivalent) were permitted during screening and for weeks 1 to 3 as needed for anxiety or insomnia, but not within 8 h prior to any psychiatric assessments. |
|  | Control: 165  Placebo | ND |  |
| **Suppes**  **(2016)** | Intervention: 180  Lurasidone | ND | Treatment with anticholinergic agents, propranolol, or amantadine was permitted as needed for movement disorders. As needed treatment with lorazepam (≤ 2 mg/d) for anxiety, or with eszopiclone (≤ 3 mg/d), temazepam (≤ 30 mg/d), or zolpidem (≤ 12.5 mg/d; for sleep) was permitted. Concomitant treatment for movement disorders, anxiety, or insomnia was not permitted within 8 h prior to any psychiatric assessments. |
|  | Control: 176  Placebo | ND |  |

LTG, lamotrigine; ND, not described
